# Resolving tricky nodes in the tree of life through amino acid recoding

**DOI:** 10.1016/j.isci.2022.105594

**Published:** 2022-11-15

**Authors:** Mattia Giacomelli, Maria Eleonora Rossi, Jesus Lozano-Fernandez, Roberto Feuda, Davide Pisani

**Affiliations:** 1 Bristol Palaeobiology Group, School of Biological Sciences, University of Bristol, Life Sciences Building, Tyndall Avenue, Bristol, BS8 1TQ, UK; 2 Bristol Palaeobiology Group, School of Earth Sciences, University of Bristol, Life Sciences Building, Tyndall Avenue, Bristol BS8 1TQ, UK; 3 Department of Genetics, Microbiology and Statistics, & Biodiversity Research Institute (IRBio), Faculty of Biology, University of Barcelona, Barcelona, Spain; 4 Department of Genetics and Genome Biology, University of Leicester, Leicester, UK

**Keywords:** Biological sciences, Evolutionary biology, Phylogenetics

## Abstract

Genomic data allowed a detailed resolution of the Tree of Life, but “tricky nodes” such as the root of the animals remain unresolved. Genome-scale datasets are heterogeneous as genes and species are exposed to different pressures, and this can negatively impacts phylogenetic accuracy. We use simulated genomic-scale datasets and show that recoding amino acid data improves accuracy when the model does not account for the compositional heterogeneity of the amino acid alignment. We apply our findings to three datasets addressing the root of the animal tree, where the debate centers on whether sponges (Porifera) or comb jellies (Ctenophora) represent the sister of all other animals. We show that results from empirical data follow predictions from simulations and suggest that, at the least in phylogenies inferred from amino acid sequences, a placement of the ctenophores as sister to all the other animals is best explained as a tree reconstruction artifact.

## Highlights

• Recoding improves accuracy when the amino acid alignment cannot be adequately modeled
• Recoding increases computational speed and can help achieving a greener phylogenomics
• There is no evidence that recoding might induce tree reconstruction artifacts
• Comb jellies are unlikely to represent the sister of all the other animals

## Graphical abstract

**Figure:**
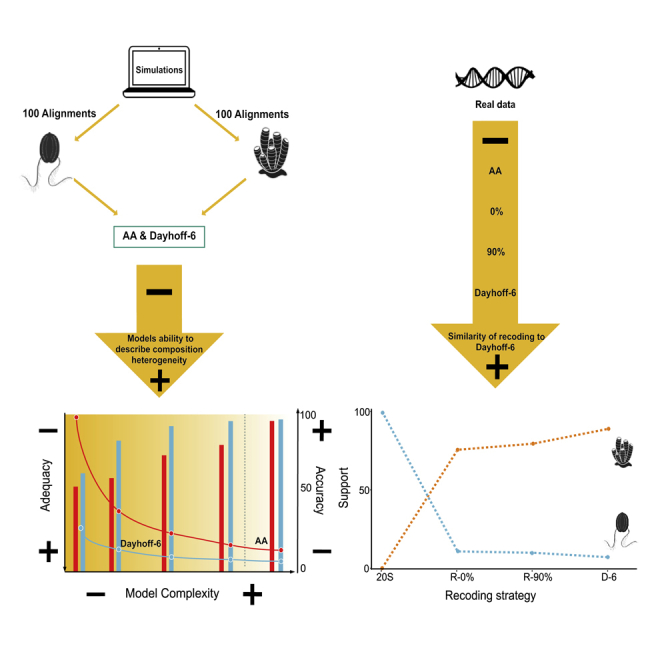

## Introduction

Genome-scale (i.e., phylogenomic) datasets have enormously increased our understanding of the Tree of Life.^1^^,^^2^^,^^3^^,^^4^ Yet, “tricky nodes” remain that are proving difficult to resolve, such as the root of the bacterial, archaeal, plant and animal trees.^5^^,^^6^^,^^7^^,^^8^^,^^9^ For example, at the root of the animal tree, it is still unclear whether the sponges (Porifera) or the comb jellies (Ctenophora) are the sister to all other metazoans,^10^^,^^11^^,^^12^^,^^13^^,^^14^^,^^15^^,^^16^^,^^17^^,^^18^^,^^19^^,^^20^ with implications reaching areas as different as developmental biology and paleoecology.^21^^,^^22^

Compositional heterogeneity is an important confounding factor in phylogenomics, and the use of evolutionary models, such as the Category (CAT^23^) and the Node Discrete Compositional Heterogeneous (NDCH^24^) model, that can respectively account for the fact that amino acids have different frequencies at different sites (hereafter referred to as across-site compositional heterogeneity) and in different species (hereafter referred to as across-lineage compositional heterogeneity) are gaining traction.^4^^,^^25^ However, even these models fail to adequately describe (i.e., fit) the heterogeneity (both across sites and lineages) of datasets designed to resolve tricky nodes in the Tree of Life.^11^

**Figure 1:**
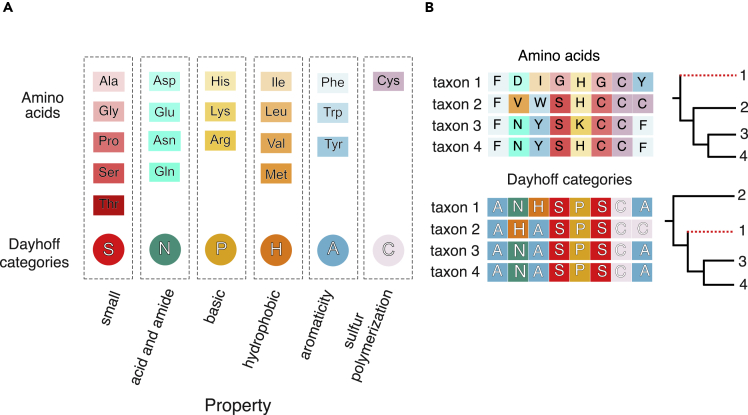
Recoding data The six-bin Dayhoff-6 recoding scheme,^33^ see STAR methods, is the most widely used recoding strategy, and is the one primarily tested in this study. Dayhoff-6 recoding partitions amino acids into six differently sized bins (see STAR methods), based on how frequently they are expected to exchange with each other. (A) The bins of the Dayhoff-6 scheme and the biochemical properties of the amino acids in each bin. (B) An exemplar amino acid dataset and its Dayhoff-6 recoded representation. Dayhoff-6 recoding is achieved by replacing, in multiple sequence alignments, one letter amino acid codes with one letter codes representing the bin where the considered amino acid is clustered.

Amino acid recoding, hereafter simply referred to as recoding (see Figure 1 and STAR Methods) was initially used to reduce the across-lineage compositional heterogeneity and saturation of amino acid datasets.^26^^,^^27^^,^^28^^,^^29^ However, recoding has also been shown to reduce across-site compositional heterogeneity ^11^ and this approach has been widely applied, frequently in combination with across-site and/or across-lineage compositionally heterogeneous models, to attempt resolving challenging nodes in the Tree of Life. Other applications of recoding can be found in the literature, e.g., to test inadequate fit of models to nucleotide data.^30^ To demonstrate the popularity of recodings, a list of more than 100 papers that used these methods has recently been published,^31^ but in a context where it was argued that recoding cannot improve phylogenetic accuracy. Although some arguments against the use of recoding have been rebutted,^32^ it has recently been suggested that recoding can only strips information from the data.^13^ This claim remains unchallenged, and it is still unclear whether recoding, in some cases, might reduce accuracy.^32^

We investigated whether recoding can improve phylogenetic accuracy using simulated alignments^12^ and three empirical datasets.^5^^,^^19^^,^^20^ We found that recoding, in the largest majority of cases, had a positive effect on phylogenetic accuracy in simulations. We show that the extent to which recoding improved accuracy correlated with the extent to which recoding decreased the Z-scores of Posterior Predictive Analyses (PPA – STAR methods) performed to test whether the model used adequately described the data. Contrary to previous conjectures,^13^ we found no evidence that recoding might cause the emergence of artifactual (i.e., incorrect but highly supported) relationships, and we therefore conclude that, at the least in our simulations, recoding is conservative because, at worst, it seems to reduce support for true clades. We show that results from three empirical datasets^5^^,^^19^^,^^20^ compare well with those from simulated data, suggesting that recoding can help resolve tricky nodes in the Tree of Life, and that a placement of the ctenophores as the sister of all the other animals, in analyses of amino acid sequence data, is best explained as a tree reconstruction artifact. Finally, we note that recoding substantially improves computational efficiency. Accordingly, we suggest that recoding has potential as a ready available tool that can be added to the list of techniques available to achieve a greener phylogenomics.^34^^,^^35^

## Results

### Recoding improves accuracy with phylogenomic scale datasets

We studied whether recoding improved phylogenetic accuracy using 200, simulated, across-site compositional heterogeneous alignments of 30,000 sites and 20 taxa.^12^ A graphical representation of our complete analytical pipeline is reported in Figure S1. The parameters of the substitution model used to simulate the data were estimated from a published superalignment^18^ under two alternative trees (Figure S2). The first tree represents a metazoan phylogeny where the ctenophores are the sister of all the other animals – Ctenophora-sister hypothesis.^10^^,^^17^^,^^19^^,^^20^^,^^36^ The second assumes that the sponges are the sister of all the other animals – Porifera-sister hypothesis.^11^^,^^14^^,^^15^^,^^16^^,^^18^ When data are simulated under Ctenophora-sister two long branches (Ctenophora and the outgroups) are on one side of the internal, target branch (Figure S2), and two short branches (Porifera and the branch leading to the other animals) are found on the other side. Ctenophora-sister therefore represents the case when the simulation target is a tree in the “Farris zone”.^12^ When Porifera-sister is the simulation target, a short and a long branch are found on both sides of the internal, target branch (Figure S2). Porifera-sister represents the case when the simulation target is a tree in the “Felsenstein zone”.^12^ One hundred datasets simulated under each topology (STAR methods for details) were analyzed in a Bayesian framework as both amino acid and Dayhoff-6 (Figure 1 and STAR methods) recoded data. Sensitivity tests were performed to evaluate whether our experimental choices affected our results – STAR methods and Figure S1.

We started our analyses using a CAT-based^23^ across-site compositionally heterogeneous model with 10 site-frequency categories. This model was combined with a Poisson (F81^37^) process that assumes that all amino acids have the same exchangeability rate, and a Gamma distribution^38^ to account for across-site heterogeneity of replacement rate – STAR methods. This is the relatively simple nCAT10-F81 + G model, which will be hereafter referred to as nCAT10. We then increased the number of site-frequency categories to 30, 60, 120 and 240 (defining nCAT30-F81 + G to nCAT240-F81 + G models; hereafter referred to as nCAT30 to nCAT240) to progressively better account for across-site compositional heterogeneity. After that, we performed analyses using the well-known CAT-F81 + G (hereafter CAT^23^) model which estimates the number of site-frequency categories necessary to best describe the across-site compositional heterogeneity of the data during tree search. We then tested a General Time Reversible (GTR + G; hereafter GTR) model that, differently from the CAT-based models described above, does not account for across-site compositional heterogeneity but models the variation in exchange rate among amino acids.^39^ Finally, we tested the CAT-GTR + G (hereafter CAT-GTR) model, which combines the properties of the CAT and GTR models and accounts for both across-site compositional heterogeneity and the heterogeneity of the amino acid replacement process.

**Figure 2:**
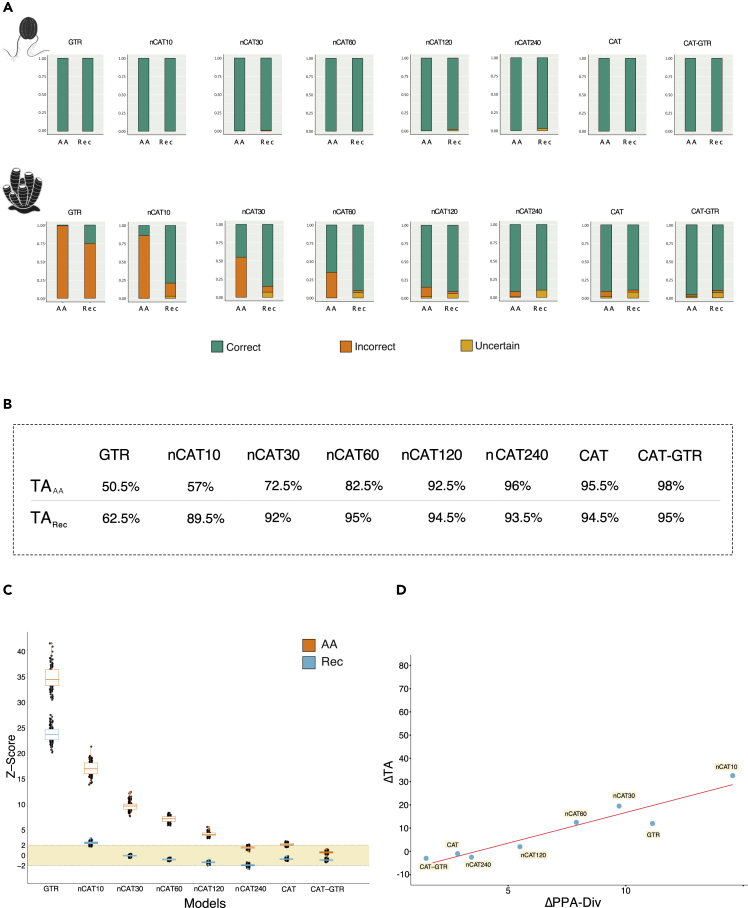
Recoding the data improves accuracy when the model fails to fit the amino acid alignment (A) Accuracy of amino acids and recoded data as models that can account for more across-sites compositional heterogeneity are used. (B) Table summarizing the Total Accuracy (TA) for amino acids and recoded data under each model. TA is calculated (from the values in A) as the percentage of accurate trees (see STAR methods) under both Porifera- and Ctenophora-sister. (C) Change in the fit (expressed as Z-scores) of the model to the data (estimated using PPA-Div) as models that can account for more across-sites compositional heterogeneity are used. In Orange amino acid datasets; in Blue recoded datasets. (D) Correlation between the difference in Z-scores achieved by each considered model on the amino acid and recoded datasets (*δ*PPA-Div), against the difference in TA achieved before and after recoding (*δ*TA). See Figures S4–S7 for sensitivity tests showing that our conclusions would not have changed if we used Maximum Likelihood instead of Bayesian analyses, if we run our Bayesian analyses 8,000 more generations (convergence was achieved before 1000 cycles), if we used a more stringent threshold to define success (PP = 0.95 instead of PP = 0.5), and if we used alternative recoding schemes (SR6 and KGB6 see STAR methods for details) instead of Dayhoff-6.

When Ctenophora-sister is the simulation target (i.e. the “Farris zone” case), the true tree was recovered with a frequency close to 100% irrespective of model and data type, i.e. Amino acids (AA) and recoded (Rec) data (Figure 2A). Differently, when Porifera-sister is the simulation target (i.e., the “Felsenstein zone” case), model and data type have a strong impact on accuracy (Figure 2A). To account for this topology-dependency, for each data type and model we calculated its Total Accuracy (TA_AA_ and TA_Rec_) as the percentage of accurately inferred trees under both topologies with a minimal posterior probability of 0.5 (see STAR methods for our definition of accuracy and Figure 2B). We used Posterior Predictive Analysis (PPA) of character state Diversity (PPA-Div – STAR methods for details and interpretation) to quantify how well different models describe the across-site compositional heterogeneity of the amino acid and recoded data.

The extent to which recoding improved accuracy and reduced PPA-Div scores seem related (Figures 2A, 2B, and 2C). The CAT-based model with the highest PPA-Div_AA_ scores (indicating the poorest fit to the amino acid data) was nCAT10 (Average PPA-Div_AA_∼ 17; Figure 2C). This model achieved much lower PPA-Div_Rec_ scores (Average PPA-Div_Rec_∼ 2.5) and emerged as the model for which recoding most strongly improved accuracy (TA_AA_ = 57%; TA_Rec_ = 89.5%; Figures 2A and 2B – a 32.5% improvement). At the other end of the spectrum, CAT-GTR achieved the lowest PPA-Div_AA_ scores (Average PPA-Div_AA_ = 0.6 – Figure 2C) indicating that this model adequately described the simulated amino acid data. CAT-GTR achieved PPA-Div_Rec_ scores comparable to those achieved by the amino acid data (Average PPA-Div_Rec_ ∼ −0.9), and emerged as the model that, with recoded data, had the worst (despite still very good) relative performance (TA_AA_ = 98%; TA_Rec_ = 95%; Figure 2B). CAT, which for our simulated datasets estimated on average 277 site-frequency categories (SD = 28.23), and nCAT240 were also found to have very low PPA-Div_AA_ scores (Figure 2C), indicating that they adequately described the amino acid data. As in the case of CAT-GTR, the estimated PPA-Div_Rec_ scores of CAT and nCAT240 (Figure 2C) were comparable to those obtained using amino acid data, and as in the case of CAT-GTR, recoding did not improve accuracy under these two models (Figures 2A and 2B). The other CAT-based models (nCAT30 to nCAT120) had PPA-Div_AA_ and PPA-Div_Rec_ scores falling between the extremes defined by nCAT10 on one side, and CAT-GTR, CAT, and nCAT240 on the other (Figure 2C). For these models, accuracy improved upon recoding (Figures 2B and 2D), with the improvement becoming less marked as the ability of the model to describe the simulated amino acid data increased, and PPA-Div_AA_ and PPA-Div_Rec_ scores became more similar (Figures 2A, 2B, and 2C). PPA-Div scores for GTR (Average PPA-Div_AA_ = 43.6; Average PPA-Div_Rec_ = 30.2) indicated that this model fits both amino acid and recoded data poorly. However, PPA scores achieved by GTR when the data are recoded are lower than those achieved by the amino acid data. As expected, also under GTR, recoding improved accuracy. However, the improvement was only 7% (Figure 2B), and it was achieved in a context where an artifactual tree was still more likely to be inferred than the target tree when Porifera-sister was assumed to be true. This result is consistent with the poor fit of GTR to both the amino acid and recoded data.

To clarify the relationship between changes in PPA-Div Z-scores and accuracy, for each model we compared *δ*PPA-Div (|PPA-Div_AA_| minus |PPA-Div_Rec_|) and *δ*TA (TA_Rec_ minus TA_AA_). In PPA analyses smaller absolute Z-scores usually identify a better absolute fit of the model to the data,^40^ and we found a strong correlation (R^2^ = 0.90) indicating that recoding seems to improve accuracy when PPA-Div_AA_ is large (i.e., the model does not adequately describe the across-site compositional heterogeneity of the amino acid data), and PPA-Div_rec_ is small (the model seems to adequately describe the across-site compositional heterogeneity of the recoded data). Notably, *δ*TA was negative (i.e., amino acids were more accurate than recoding – Figure 2D) for models that already adequately described the across-site compositional heterogeneity of the amino acid alignments (CAT, nCAT240, and CAT-GTR – see Figure 2C). These results are consistent with the hypothesis that recoding increased accuracy when it improved the fit of the data to the model. This behavior can be expected because, when a model has a good absolute fit to the amino acid data, there should be no model violations left for the recoding to mask. Therefore, with our simulated data, we would expect recoding to improve accuracy only for GTR, nCAT10, nCAT30, nCAT60 and nCAT120. This is because according to PPA-Div_AA_ scores these models do not fit the amino acid data well. However, lower PPA-Div scores are also expected when the size of the state space of a dataset is reduced, which is the case when 20-state amino acid data are recoded using the 6-state Dayhoff-6 scheme (Figure 1 and STAR methods). Although we address this problem in the next section using random recodings, to be conservative (given the size difference between the amino acid and recoded alphabets), we only conclude that our results are consistent with the hypothesis that recoding improved accuracy when it improved the fit of the data to the model but cannot prove it. However, the strong correlation between *δ*PPA-Div and *δ*TA in Figure 2D clearly indicates that recoding the data invariably had a positive effect on the accuracy of models that did not adequately describe the across-site compositional heterogeneity of the amino acid alignments.

Recoding is expected to reduce saturation.^11^^,^^29^ We estimated saturation levels (STAR methods) for our amino acid and recoded data, finding recoded alignments to be less saturated. However, the level of saturation observed for the recoded data under nCAT30, nCAT60, nCAT120, nCAT240, CAT and CAT-GTR was statistically indistinguishable – the distributions overlapped (Figure S3). Accordingly, the reduced saturation of the recoded data cannot explain differences in TA observed for these models, suggesting that a reduction in saturation is not the main mechanism by which recoding improves accuracy.

We investigated whether our methodological assumptions might have affected our results. We tested the effect of using Maximum Likelihood instead of Bayesian analysis, the effect of increasing the number of generations in our Bayesian analyses, the use of alternative 6-state recoding strategies, and the use of a more stringent criterion (higher support) to define accuracy (see STAR methods and Figures S4–S7), finding no significant difference.

**Figure 3:**
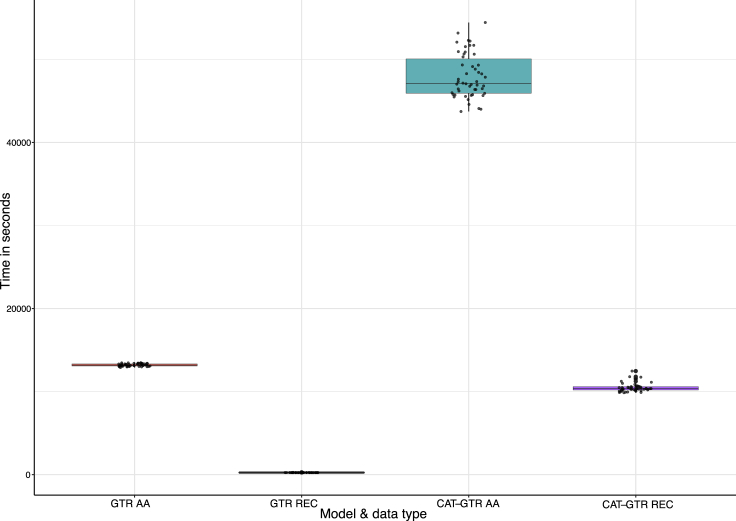
Recoding as a tool in green phylogenomics Time taken to complete 50 GTR and CAT-GTR analyses of amino acid and recoded datasets. Recoded analyses are invariably completed in a shorter time, with the difference becoming significantly more marked when using the complex CAT-GTR model. Given the high accuracy of recoded analyses this result suggests that recoding could play a significant role in the development of “green phylogenomics”.

We recorded the time needed to complete analyses of amino acid and recoded data, finding that recoding improves computational efficiency and significantly reduces the time needed to complete the analysis (Figure 3). This result suggests that recoding has significant potential as a ready available tool for “green phylogenomics”.^34^^,^^35^ This is because, by speeding up analyses, recoding reduces emissions without compromising phylogenetic accuracy (Figure 2D).

### Randomization experiments suggest that recoding reduce across-site compositional heterogeneity and improves the fit of the data to the model

Recoding is based on the theoretical prediction that there should be more within-bin (i.e. same amino acid category) than across-bin (i.e. different amino acid category) substitutions (STAR methods and Figures 1 and S8), and that the within-bin substitutions contribute more lineage-specific and site-specific compositional heterogeneity than across-bin substitutions (see STAR methods). Randomly generated recoding schemes^13^^,^^29^ can be defined (see STAR methods for details). Such schemes should be expected to reduce compositional heterogeneity (both across-sites and lineages) less than empirical recodings that account for biochemical and biophysical properties of amino acids such as Dayhoff-6 (Figure 1 and STAR methods). This is because random recodings will not necessarily mask substitutions between amino acids that replace frequently among each other due to their similar biochemical and biophysical properties^13^ – STAR methods and Figure S8. Accordingly, we can expect that randomly recoded datasets should fit models worse than datasets recoded using Dayhoff-6 (the recoding used in our study). Furthermore, trees inferred from randomly recoded datasets should be expected to have higher tree length (random recodings will mask less substitutions) and be less accurate than trees inferred from datasets recoded using Dayhoff-6.

We used 200 6-state random recoding schemes based on the binning-structure of Dayhoff-6 (Figure 1 and STAR methods for details) to test whether within-bin substitutions are more abundant and contribute more compositional heterogeneity than across-bin substitutions, and to test whether Dayhoff-6 improves fit and accuracy more than random recodings. It should be noted that (I) randomly recoded datasets are still expected to infer meaningful trees, as they only mask a random subset of substitutions within an alignment, with the remaining substitutions being unchanged (Figure S8 for a visual representation of this phenomenon). (II) There is only a limited number of ways in which 20 amino acids can be allocated to six bins of the same size of those of Dayhoff-6, and random recoding schemes are therefore expected to frequently retain some random similarity to Dayhoff-6 (see STAR methods). (III) Because the size of the character state space of randomly generated recodings and Dayhoff-6 is the same, PPA-Div scores from datasets recoded using Dayhoff-6 and our 200 random recodings schemes should be comparable – differently from the case of PPA-Div scores of recoded and amino acid data (see previous section). Based on points 1 to 3 above, we can predict that if recoding works as expected, random recodings of greater residual similarity to Dayhoff-6 should mask more substitutions, achieve greater accuracy, and fit the data better than random recodings that retained less residual similarity to Dayhoff-6. We generated 100 random recoding schemes with 90% residual amino acid similarity to Dayhoff-6 (90%-recodings), and 100 random recoding schemes with no residual amino acid similarity to Dayhoff-6 (0%-recodings) – Figure S8 and STAR methods.

**Figure 4:**
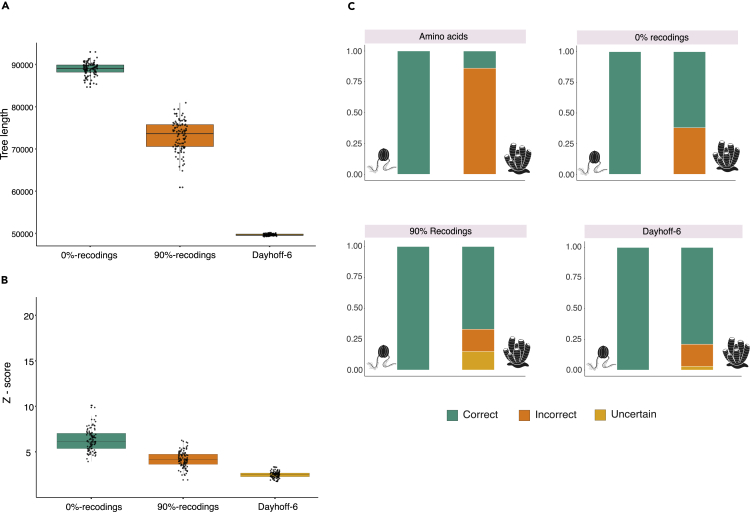
Dayhoff-6 outperforms random recoding schemes (A) Boxplot representing the distribution of tree lengths for 0%-recoded, 90%-recoded, and Dayhoff-6 recoded dataset. (B) Comparison of PPA-Div scores for 0%-recoded, 90%-recoded, and Dayhoff-6 recoded datasets. The figure indicates that PPA-Div scores of Dayhoff-6 recoded data are significantly better than PPA-Div scores from 0%-recoded data – the distributions do not overlap. (C) A comparison of the TA values achieved by amino acids data, 0%-recoded, 90-%recoded and Dayhoff-6 recoded data, indicating that Dayhoff-6 outperforms both amino acids and randomly recoded data.

Trees inferred from Dayhoff-6 recoded data had an average tree length of 49,683.18 parsimony steps. Trees inferred from 90%-recodings were 72,390.12 steps long on average, and trees inferred from 0%-recodings had an average tree length of 88,046.08 steps. These results indicate that Dayhoff-6 masks ∼39% and ∼23% more substitutions than 0%- and 90%-recodings (Figure 4A), confirming that amino acids that are classified in the same bin by Dayhoff-6 substitute more frequently than amino acids that are placed in different bins. PPA-Div scores obtained from Dayhoff-6 recoded datasets (Average PPA-Div_Rec_∼ 2.5; SD = 0.33) are significantly better (the distributions do not overlap) than PPA-Div scores from 0%-recoded datasets (Average PPA-Div_0%_∼ 6; SD = 1.2), whereas PPA-Div scores from 90%-recoded datasets (Average PPA-Div_90%_∼ 4; SD = 0.87) overlap with both PPA-Div_Rec_ and PPA-Div_0%_ (Figure 4B). Finally, the extent to which random recodings improved accuracy (TA_0%_ = 81%; TA_90%_ = 83.5%) was found to depend on their similarity to Dayhoff-6 (TA_Rec_ = 89.5%) – Figure 4C. These results show that Dayhoff-6 performs better than random recodings and that Dayhoff-6 recoded data have a better fit to the model than randomly recoded data. These results suggest that Dayhoff-6 helps improving the fit of the data to the model.

### The performance of recoding improves as more sites and taxa are used

**Figure 5:**
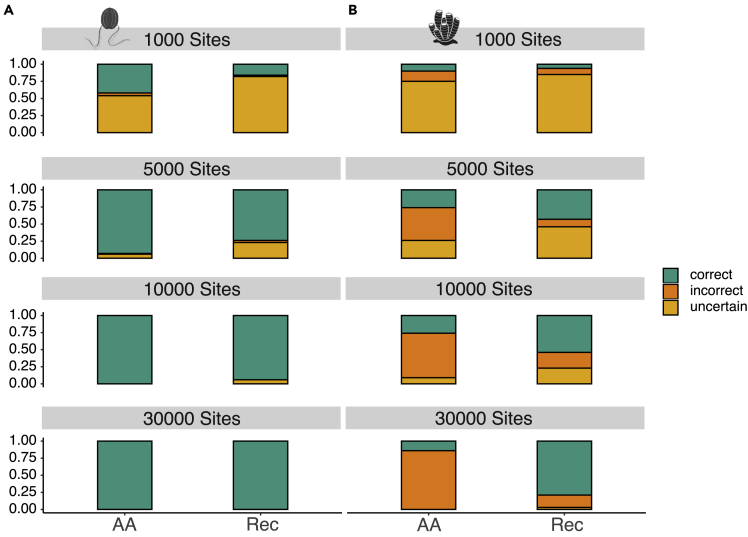
Accuracy of recoded data increases with alignment size (A) Accuracy of amino acid and recoded datasets of 1,000, 5,000, 10,000 and 30,000 sites analyzed under nCAT10, when the generating tree assumes Ctenophora-sister to be true. (B) Success rate of amino acid and Dayhoff-6 datasets of 1,000, 5,000, 10,000 and 30,000 sites analyzed under nCAT10, when the generating tree assumes Porifera-sister to be true. Analyses performed in Phylobayes. In Green: Correct trees; Dark Orange: Incorrect Trees; Light Orange: Uncertain trees.

Our random recoding experiments reject the view^13^ that recoding merely strip information from the data as this would be incompatible with the results in Figure 4. However, every recoding strategy, including the widely accepted recoding of nucleotides into amino acids, incurs a cost in the form of an information loss^41^ (STAR methods). We estimated (STAR methods) that the information lost from our simulated alignments when the data were recoded from amino acids to Dayhoff-6 was ∼49%, and that this loss was homogeneously distributed across the branches of the target topology (see Figure S9). Accordingly, we hypothesized that alignment length can be expected to have an impact on the performance of recoded datasets, as longer alignments will have more substitutions (everything else being equal) and should therefore be expected to be more informative. To test this hypothesis, we analyzed alignments with 20 taxa and 1,000, 5,000, 10,000 and 30,000 sites. We found that with 1,000 site alignments, amino acids performed best, but in a context where both data types had very poor accuracy (TA_AA-1000_ = 26% and TA_Rec-1000_ = 11%, Figure 5). With 5,000 site alignments, accuracy improved for both data types with amino acids doing marginally better (TA_AA-5000_ = 59.5% and TA_Rec-5000_ = 58.5%, Figure 5). These results agree with what was observed in previous studies.^31^^,^^32^ Finally, with alignments of 10,000 and 30,000 sites, we found recoded data to outperform amino acids (TA_AA-10,000_ = 63%, TA_Rec-10,000_ = 74%, and TA_AA-30,000_ = 57%, TA_REC-30,000_ = 89.5%; Figure 5), consistent with the results of Foster et al.^32^ With reference to taxon sampling (Figure S10), we found that accuracy increases as more taxa are included in the dataset, as it is common when the data are affected by attraction artifacts.^42^^,^^43^

### Recoding of empirical datasets can help resolve tricky nodes

The target trees used in our simulations represent the phylogenetic uncertainty at the root of the animal tree (Ctenophora-sister versus Porifera-sister hypothesis). The alignments we used were simulated under a CAT-LG + G model (hereafter CAT-LG) that was parameterized using values estimated from a dataset designed to study early animal relationships.^18^ We thus applied the knowledge gained from our simulations to three, different (i.e. independently assembled) datasets that were also designed to address the same problem^5^^,^^19^^,^^20^ – hereafter referred to as Whelan2015, Whelan2017, and Laumer2019). To make sure results from the simulated and the empirical data were comparable, we evaluated whether the across-site compositional heterogeneity of the three empirical datasets could have been generated by a process that could be described using a model that is not too dissimilar to CAT-LG. To do so we used PPA-Div_AA_ to test how well CAT-LG can describe the across-site compositional heterogeneity of Whelan2015, Whelan2017, and Laumer2019. PPA-Div_AA_ indicates that CAT-LG adequately describes the across-site compositional heterogeneity of Laumer2019 (PPA-Div_Laumer2019_ = 1.55), but not that of Whelan2015 and Whelan2017 (PPA-Div_Whelan2017_ = 4.69; PPA-Div_Whelan2015_ = 4.64). However, PPA-Div_AA_ scores for Whelan2015 and Whelan2017 are rather small (compare with those in Figure 2C) suggesting that models that are not too different from CAT-LG should be able to adequately describe Whelan2015 and Whelan2017.

**Figure 6:**
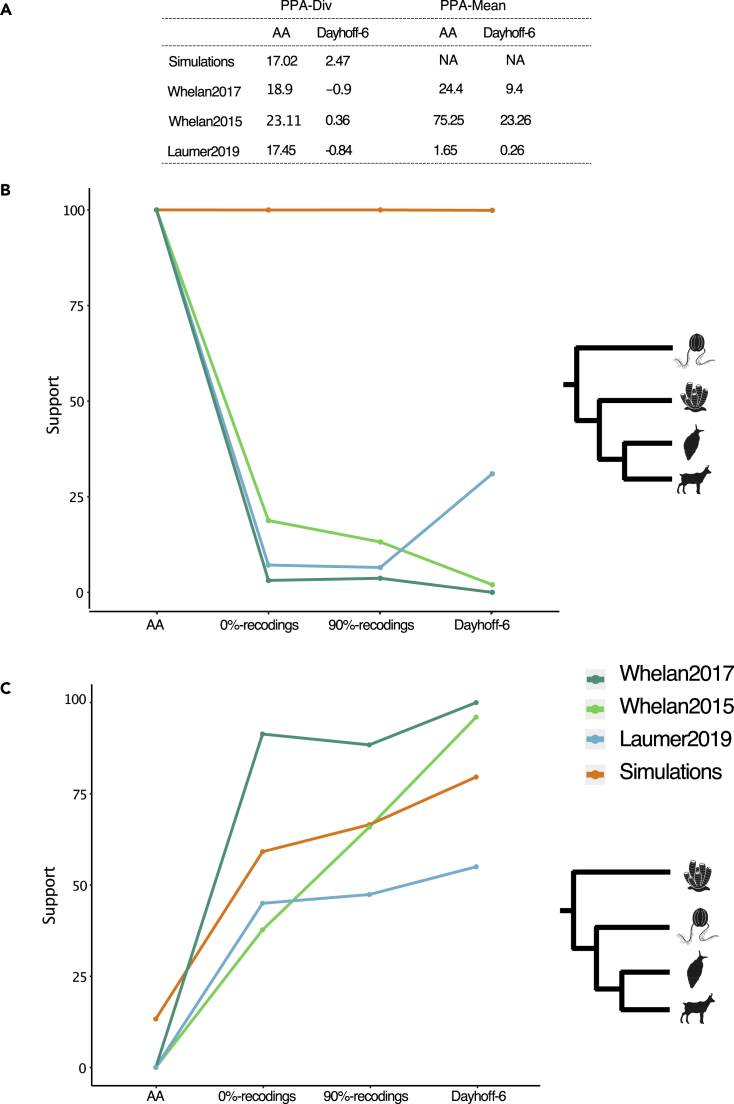
Results from empirical datasets follow predictions from simulations (A) PPA-Div and PPA-Mean scores for all three empirical datasets when the alignments are analyzed as amino acids and Dayhoff-6 recoded data. Average PPA scores for our simulated data are also reported (under nCAT10), indicating that PPA scores achieved for the simulated data under nCAT10 are comparable to those achieved under nCAT60 for the empirical datasets. (B) Support for Ctenophora-sister as different data types (amino acids, 0%-recodings, 90%-recodings, and Dayhoff-6) are used. (C) Support for Porifera-sister as different data types (amino acids, 0%-recodings, 90%-recodings, and Dayhoff-6) are used. In Orange: reference support values obtained for the two considered clades in our simulations. Light Green: Whelan2015; Dark Green Whelan2017; Blue Laumer2019. Note: Support values for 0%-recodings and 90%-recodings represent average values calculated over all random recoding generated.

We then analyzed Laumer2019, Whelan2015, and Whelan2017 as amino acid alignments, and using 0%-recodings, 90%-recodings, and Dayhoff-6. For all analyses the nCAT60 model was used. This was done to maintain comparability between our results and those of Li et al.^13^, who conjectured that recoding only strip signal from the data. For all datasets we estimated PPA_AA_ and PPA_Rec_, as well as support values for both Porifera- and Ctenophora-sister for all four data types. We used both PPA-Div and PPA-Mean (STAR methods) to test whether nCAT60 adequately describes the heterogeneity of these datasets, as empirical data usually display compositional heterogeneity across both sites and taxa. PPA-Div_AA_ (Figure 6A) indicates that the across-site compositional heterogeneity of the three amino acid datasets is described very poorly by nCAT60. PPA-Mean_AA_ (Figure 6A) indicates that across-lineage compositional heterogeneity is unlikely to be a problem for Laumer2019, but nCAT60 describes very poorly the across-lineage compositional heterogeneity of Whelan2015 and Whelan2017. PPA-Div_Rec_ and PPA-Mean_Rec_ indicate that nCAT60 adequately describes the across-site compositional heterogeneity of the three recoded datasets and that also when using Dayhoff-6 across-lineage compositional heterogeneity should not be a problem for Laumer2019. For Whelan2015 and Whelan2017, PPA-Mean_Rec_ indicates that recoding reduced across-lineage compositional heterogeneity, but the fit was still poor. nCAT60 is not an across-lineage compositional homogeneous model. Accordingly, its inability to model the across-lineage compositional heterogeneity of the recoded versions of Whelan2015 and Whelan2017 was expected, given the high PPA-Mean_AA_ scores achieved by these datasets (Figure 6A). However, the across-lineage compositional heterogeneity of these datasets is still much better described on the recoded data.

For all three datasets support for Ctenophora-sister was maximal (PP = 1) when the data were analyzed as amino acids (Figure 6B). In the cases of Whelan2015 and Whelan2017 support for Ctenophora-sister progressively decreased, becoming minimal (PP_Whelan2015_ = 0.02 and PP_Whelan2017_ = 0) when the data are Dayhoff-6-recoded. In the case of Laumer2019 minimal support for Ctenophora-sister was achieved using 90%-recodings (PP = 0.06). Support for this clade increased to PP = 0.31 underDayhoff-6, but PP = 0.31 is still within the range of what is expected for random resolutions. For all three datasets, support for Porifera-sister followed the opposite trend, being minimal (PP = 0) for amino acid datasets, and maximal (PP_Laumer2019_ = 0.55; PP_Whelan2015_ = 0.96; PP_Whelan2017_ = 1) under Dayhoff-6 (Figure 6C). The lower discriminatory power of the recoded version of Laumer2019 (Ctenophora-sister PP = 0.31 and Porifera-sister PP = 0.55) may be a consequence of this dataset being less informative than Whelan2017 and Whelan2015. Laumer2019 had an average of ∼5 substitutions per site (see STAR methods) while Whelan2015 and Whelan2017 had an average of ∼7 substitutions per site, and recoding can be expected to work best with more informative datasets that better absorb the loss of information caused by its application (see above). Irrespective of that, the trend observed for the changes in support levels for Ctenophora-sister and Porifera-sister, are comparable across the three datasets. We plotted (Figures 6B and 6C) average support values observed for our simulated data under nCAT10 (the model that in simulations achieved PPA-Div_AA_ scores comparable to those obtained using nCAT60 with the empirical data). For all three datasets, changes in support values for Ctenophora-sister strongly disagree with what is observed in simulations where Ctenophora-sister is true. On the other hand, changes in support levels for Porifera-sister follow the pattern observed in simulations where Porifera-sister was true.

Our simulations suggest that when the model used does not adequately describe the compositional heterogeneity of the amino acid data (as in the case of the three considered empirical datasets), recoding improves phylogenetic accuracy. Li et al.^13^ stated that if recoding was a valid approach to improve phylogenetic accuracy, it should be expected that if a clade is true it should be recovered in analyses using an empirical recoding such as Dayhoff-6, but not in analyses using random recodings. Our random recoding simulations show that this conjecture is only partially correct. It is correct that Dayhoff-6 outperforms random recodings, but random recodings still improve phylogenetic accuracy, a result that is not novel,^29^ and the performance of random recodings depends on their similarity to Dayhoff-6 (see above), a result that is not unexpected. Accordingly, we reformulate Li et al.^13^ conjecture to state that: within the limit of utility of recoding methods (e.g., when the model does not describe the compositional heterogeneity of the amino acid data well – see above), true clades should be expected to achieve progressively better support as random recodings more similar to Dayhoff-6 are used. For artifactual clades, the opposite trend should be expected. Analyses of our three empirical datasets consistently find that support values for Porifera-sister follow the trend expected for true clades while support values for Ctenophora-sister follow the trend expected for artifactual clades.

## Discussion

### Recoding is justified

The use of recoding has been justified based on its predicted ability to mask substitutions that are *a-priori* expected to contribute more compositional heterogeneity and saturation – i.e., within-bin substitutions.^11^ Using our simulated data and random recoding we could confirm that: (I) within-bin substitutions are more frequent than across-bin substitutions, as Dayhoff-6 removes more substitutions than random recodings. (II) Recoding seems effective at improving fit and reducing across-site compositional heterogeneity as PPA-Div scores of alignments recoded using Dayhoff-6 are better than those obtained using random recodings. (III) Recoding improves accuracy when there is a sizable difference in *Z*-score between the recoded and the amino acid data (Figures 2C and 2D). Although this difference in Z-scores can be partially attributed to the smaller size of the recoded alphabet, random recoding experiments suggest that as we mask within-bin substitutions (and recoding amino acid data masks within-bin substitutions – Figures S8 and S9), across-site compositional heterogeneity decreases while fit and accuracy increase (Figure 4). We therefore suggest that recoding can improve phylogenetic analyses in two ways. First, by making the parameter space smaller, recoding makes model parametrization easier and computationally more efficient, given the unmasked data. Second, by simplifying the data (masking sites with a high proportion of multiple substitutions), recoding improves the ability of the model to describe the compositional heterogeneity of the data. That is, recoding can improve the fit of the data to the model, which is crucial to improve accuracy.^40^^,^^44^

A previous investigation attempted comparing recoding and random recoding schemes and concluded that recoding did not perform better than random.^13^ However, that study used only four random recoding schemes, and may have lacked statistical power. Our analyses used 200 random recoding schemes of different similarity to Dayhoff-6 (0%- and 90%-recodings), and robustly establishes that both real and to a lesser extent random recodings can improve accuracy, as even 0%-recodings outperform amino acids (TA_0%_ = 81%; TA_AA_∼ 57% – compare Figures 2 and 4) in our simulations. This result might seem counterintuitive, and its full interpretation is outside of the scope of this study. However, it should not surprise that 0%-recodings infer valid trees, given that random recoding schemes do not randomize the data, they randomly mask a subset of substitutions, with the unmasked substitutions being shared with the amino acid data (Figure S8). We hypothesize that the improved accuracy observed with 0%-recodings might be a consequence of the fact that a 6-state model is more easily parametrized (given the recoded data) than a 20-state model (given the amino acid data), a testable hypothesis.

### Recoding improves accuracy when the alignment is sufficiently long

Recoding incurs a cost in the form of an information loss (which for our simulated datasets was approximately 49%, Figure S9 and STAR methods), and our results indicate that the use of recoding should be limited to phylogenomic-scale datasets that can absorb this cost. Hernandez and Ryan^31^ investigated this problem using 1,000 to 5,000 site alignments and concluded that the use of 6-state recoding schemes (such as Dayhoff-6) cannot improve accuracy, despite finding that recodings with more states had a positive effect on accuracy. When using datasets of 1,000 to 5,000 sites we found the same result: Dayhoff-6 does not improve accuracy, indicating that there is a limit to the use of 6-state recodings that might not be useful in analyses of single genes or of small genomes (e.g. viral genomes). However, when we used phylogenomic-scale alignments (10,000 sites or more) recoding significantly improved accuracy (Figure 5), when the model used to analyze the data was not a good fit to the amino acid alignments (Figure 2D). We interpret these results to suggest that, with phylogenomic scale datasets, when the absolute fit of the model to the amino acid data is poor, the trade-off between information loss and improved fit favors recoding. Differently, when the model already adequately describes the across-site compositional heterogeneity of the amino acid data, there will be no model violations left for the recoding to aemiliorate. However, some information will still be lost when the data are recoded, tilting the trade-off balance against the use of recoding. We suggest that the mild deterioration of accuracy observed for nCAT240, CAT and CAT-GTR, and the conclusion of Foster et al.^32^ that recoding does not always increase accuracy, explained within the context of this trade-off.

Because recoding does not always improve accuracy (see^32^ and above), a criterion is necessary to guide its application. The correlation in Figure 2D suggests that the comparison of PPA_AA_ and PPA_Rec_ scores provide valuable information to decide whether the use of recoding is appropriate, even if these values do not purely record changes in fit between amino acid and recoded alignments, because they are summary statistics computed on data types with different state spaces.

Simulated datasets are abstractions of empirical data. They can be limited in the number of sites^31^ or taxa,^32^ and their heterogeneity will not perfectly match that of empirical data. For example, our simulated data are only across-site compositionally heterogeneous, whereas other studies used alignments that were only across-lineage compositionally heterogeneous.^29^^,^^31^ Based on our simulations one could erroneously conclude that recoding is redundant when using relatively well fitting models such as nCAT60, CAT and CAT-GTR. However, absolute goodness-of-fit tests^40^^,^^44^ show (see Figures 6 and ^11^) that even models such as CAT-GTR fail to adequately describe the compositional heterogeneity of empirical datasets designed to address challenging nodes in the Tree of Life. Accordingly, it can be expected that with empirical data there should be substantial scope for recoding to improve accuracy also when the best available site-heterogeneous models are used.

### Is Ctenophora-sister a tree reconstruction artifact?

Our simulations suggest that when the alignment is sufficiently long and the model used to analyze the data fails to adequately describe the compositional heterogeneity of the amino acid alignment, recoding should improve accuracy. Based on these conclusions, we moved on to exemplify the use of recoding, and tested three empirical datasets for the root of the animal tree. We chose these datasets because they are published and their taxon sampling compares well with that of the dataset used to parametrize our simulated data. It has been suggested^13^ that if recoding was a valid approach to improve phylogenetic accuracy (and according to our simulations it is), when amino acid and Dayhoff-6 datasets disagree, true clades should be recovered in analyses of Dayhoff-6 recoded dataset, but not in analyses of randomly recoded datasets. Our simulations found that the suggestion of Li et al.,^13^ as formulated, is not valid. Dayhoff-6 recoded datasets achieve greater accuracy than randomly recoded datasets, but, randomly recoded datasets perform better than amino acid datasets, with their performance depending on their residual amino acid similarity to Dayhoff-6 (see above for details). Accordingly, Li et al.’s^13^ conjecture should be rephrased to say that, in cases when recoding can be expected to improve accuracy (and Figure 6A suggests this should be the case for our empirical datasets), support for true clades should increase as the data are recoded using random recoding schemes that are progressively more similar to Dayhoff-6. For artifactual clades, the opposite trend should be expected. Results from our three empirical datasets consistently show that changes in support for Porifera-sister follow the trend expected for real clades, while support values for Ctenophora-sister follow the trend expected for artifactual clades.

The debate on the root of the animal tree has seen a diversity of analyses and data. Sponges as the sister of all the other animals is favored by morphology and gene content data.^45^^,^^46^^,^^47^ Amino acid datasets analyzed using CAT and CAT-GTR tend to switch between Porifera– and Ctenophora–sister^16^ depending on outgroup sampling. In these experiments, Porifera-sister is frequently (but not always) supported when close outgroups (not only Choanoflagellata^16^), which should reduce attraction artifacts,^11^^,^^16^^,^^18^^,^^19^^,^^20^^,^^48^ are used. A common feature of investigations of early animal relationships is that the models used to analyze the data usually fail to a greater (GTR) or lesser (nCAT60/CAT/CAT-GTR) extent to adequately describe the compositional heterogeneity of the relevant alignments (see^11^ and Figure 6). When datasets are recoded, CAT-based models (but not GTR) achieve a much better fit (see,^11^ Figures 2 and 6). These are the conditions when we can expect recoded data to improve accuracy, and CAT-based analyses of recoded data almost invariably reject Ctenophora-sister – see^49^ for an exception.

It has been claimed that analyses of sequence data supporting Porifera-sister represent either CAT-induced overparameterization artifacts, or recoding-induced signal-loss artifacts.^13^ Overparameterization in Bayesian analysis (including analyses that use CAT) has been studied,^50^^,^^51^^,^^52^^,^^53^ but no evidence was found that it can cause the emergence of tree reconstruction artifacts. Similarly, our simulations did not find any evidence that recoding can induce the emergence of tree reconstruction artifacts. Differently, recent analyses seem to suggest that the artifactual inference of Ctenophora-sister can also be caused by incomplete lineage sorting.^54^

It has been argued that we should be skeptical of Porifera-sister in analyses of sequence data because it is found only under a narrow range of models that do not fit the data better than models supporting Ctenophora-sister.^13^ However, the size of the model space supporting Ctenophora-sister is not as broad as claimed because models such as WAG, LG, JTT,^55^^,^^56^^,^^57^ etc., are just alternatively parameterized GTR matrices. If one were to consider (and explicitly name) alternative parameterizations of CAT, the range of models under which Porifera-sister is supported would similarly expand. Furthermore, none of these alternatively parametrized GTR matrices fits as well as CAT or CAT-GTR, both of which frequently support Porifera-sister,^16^^,^^58^ the support for which is not limited to using CAT – contrary to what implied by Li et al.^13^ Although it has been shown that nCAT60 (another across-site compositionally heterogeneous model) could not be distinguished in some Bayesian cross-validations from CAT,^13^ that does not imply that CAT is overparameterized and that nCAT60 should be preferred.^59^ CAT uses more frequency categories but the advantage of Bayesian methods^23^^,^^52^ is exactly that they allow the complexity of the model to be tuned to the dataset being analyzed^23^ and more adequately describe it. Modern model selection procedures should not be limited to the identification of the relative best fit model(s).^40^ Absolute goodness-of-fit tests (such as PPA) should also be used to discriminate models, including models of relative best fit.^40^^,^^44^ Absolute goodness-of-fit tests indicate that irrespective of its status as a relative co-best fit model, nCAT60 describes the across-site compositional heterogeneity of Whelan2017 (PPA-Div_nCAT60_ = 18.9) very poorly, and much worse than CAT (PPA-Div_CAT_ = 4.13). This result is consistent with those of Bujaki and Rodrigue,^59^ and indicates that contrary to Li et al.^13^, CAT should be preferred to nCAT60.

The solution to the problem of the root of the animal tree is not going to be found by counting the number of models (or studies) supporting alternative trees as suggested by Li et al.^13^ To understand phylogenetic relationships at the root of the animal tree, we must understand why, under the relative best fit model (be that CAT, CAT-GTR, or nCAT60), support switches from Ctenophora– to Porifera–sister as factors such as data type^60^ and outgroup sampling change. One hypothesis is that even current compositionally heterogeneous models struggle to adequately describe these datasets.^11^^,^^16^ In such situations, recoding can improve the fit of the data to the model, which is crucial to improve accuracy, and the analysis of recoded alignments under CAT, CAT-GTR, and nCAT60 almost invariably reject Ctenophora-sister independently of outgroup choice.^11^ We interpret these results to suggest that a placement of the ctenophores as the sister of all the other animals, at the least using sequence data, continues to be better explained as a tree reconstruction artifact.

### Conclusions

Phylogenetic accuracy of sequence data crucially depends on the fit of the model to the data.^25^ Our results suggest that recoding seems to improve the fit of models that fail to adequately describe amino acid alignments, increasing accuracy in simulations. Empirical datasets assembled to resolve tricky nodes in the Tree of Life usually display high levels of compositional heterogeneity, and available models fail to fit them.^11^ Although the development of models that could adequately fit arbitrary levels of across-site and across-lineage compositional heterogeneity^48^^,^^61^ would be welcome, efficient implementations of these models are not yet on the horizon. In the absence of such models, the analysis of recoded data remains a useful tool to study tricky nodes in the Tree of Life. At the least according to our simulations, recoding also has the advantage of drastically reducing computational time, particularly when complex models such as CAT-GTR are used, without compromising accuracy. Accordingly, recoding should be considered as a readily available tool for “green phylogenomics”.

It is not yet clear whether sponges or ctenophores represent the sister of all the other animals. Every data type has its own strengths and weaknesses, and a single line of evidence cannot resolve this controversy. Only consilience of multiple lines of evidence,^4^^,^^47^^,^^62^^,^^63^^,^^64^ e.g., sequence data, gene content data, morphology and newly emerging sources of genomic information such as chromosome tectonic,^65^ will ultimately clarify this problem. However, sequence data are the genomic data type that we better understand and for which we have better established models, and arguments based on the fit of amino acid and recoded data suggest that, according to sequence data, a placement of the ctenophores as the sister of all the other animals continue to be better explained as a tree reconstruction artifact.

### Limitations of the study

The effect of using recodings strategies with a number of bins different from 6 was not investigated. Our choice of using 6-state recodings was based on the fact that 6-state recodings are much more frequently used in the literature than other recoding schemes. However, we acknowledge that recodings with 2–19 states could be designed and should ideally be tested, as other recodings might be discovered to perform better than 6-state ones. However, even if a recoding scheme with different bin numbers could be shown to outperform the 6-state recodings used here, it will not change the fact that 6-state recoding, in our simulations, performed well. Indeed, we do not expect that our conclusions would change if such recoding strategies were used. Although our simulations were extensive, we only compared the results of our simulations to three empirical datasets that focused on the root of the animal tree. While we acknowledge that further tests on real data should be completed to further generalize our conclusions, at this time we have no reason to believe that our conclusions about the root of the animal tree would change if other empirical datasets were analysed.

## STAR★Methods

### Key resources table

**Table:** 

REAGENT or RESOURCE	SOURCE	IDENTIFIER
**Software and algorithms**		

random_recoding_generator.pl	This study	https://bitbucket.org/bzxdp/giacomelli_et_al_2022_recodings/src/master/Scripts/
compare_recodings.pl	This study	https://bitbucket.org/bzxdp/giacomelli_et_al_2022_recodings/src/master/Scripts/
Recode_to_random.pl	This study	https://bitbucket.org/bzxdp/giacomelli_et_al_2022_recodings/src/master/Scripts/
Phylobayes	N/A	https://github.com/bayesiancook
RAxML	N/A	https://github.com/stamatak

**Other**		

Simulated datasets	Kapli and Telford (2020)^12^ and This paper	https://bitbucket.org/bzxdp/giacomelli_et_al_2022_recodings/src/master/Simulated_Datasets/
Random recoding schemes	This study	https://bitbucket.org/bzxdp/giacomelli_et_al_2022_recodings/src/master/Random_recodings/
Supplementary file 1 (all results)	This study	https://bitbucket.org/bzxdp/giacomelli_et_al_2022_recodings/src/master/
Table S1 (similarity scores of random recodings and Dayhoff-6)		https://bitbucket.org/bzxdp/giacomelli_et_al_2022_recodings/src/master/

### Resource availability

#### Lead contact

Further information and requests for resources should be directed to and will be fulfilled by the Lead contact, Davide Pisani at davide.pisani@bristol.ac.uk.

#### Materials availability

This study did not generate new unique reagents.

#### Data and code availability

All the data, material and custom scripts used in this study available in Bitbucket: https://bitbucket.org/bzxdp/giacomelli_et_al_2022_recodings/src/master/. Links for individual scripts and data are reported in the key resources table. No new data were generated with this article.

### Methods details

#### Recoding strategies: A description and a logical rationale for their generalisation

Nucleotide sequences of protein coding genes can be recoded into codons or amino acid data, and phylogenomic analyses addressing tricky nodes in the Tree of Life usually use data recoded into amino acid sequences. This is because amino acids are expected to be less saturated and heterogeneous than nucleotides due to the degeneracy of the genetic code,^66^which implies that not all mutations in a nucleotide sequence correspond to an amino acid substitution. Nucleotide sequences representing protein coding genes could also be recoded using “high level recodings”,^41^ where codons (or amino acids) are grouped into bins (or categories) based on the chemical and physical properties of the amino acid they code for.

A universe of alphabets can be defined into which nucleotide sequences of protein coding genes could be recoded. These alphabets can have different cardinality, with the largest possible alphabet having 64 states (one for each possible codon), and the smallest 2 states, e.g. a coding strategy where only hydrophobic and hydrophilic amino acids/codons are distinguished (the latter is implemented in Phylobayes as Dayhoff-2). The classic 20-state (amino acid) recoding is therefore just an instance within the universe of possible recodings into which nucleotide sequences of protein coding genes could be translated. Indeed, experiments have been performed, for example, using an alternative 23-state recoding^66^ distinguishing, in addition to the 20 amino acids, the codon classes of the three 6-fold degenerate amino acids. However, these analyses showed the use of a 23-state recoding was not providing advantages over a 20-state recoding suggesting that recodings with more states are not necessarily better for resolving phylogenetic relationships.

Compared to other recodings, 20-state amino acid recording has the advantage that its use can be promptly justified on biological grounds, as it represents the primary structure of protein sequences. It is thus logical and appropriate that we consider amino acid recoding to be the default strategy to recode nucleotide sequences of protein coding genes, and our study should not be interpreted as challenging this view. However, this does not imply that we should assume *a-priori* that amino acid recoding would maximise phylogenetic accuracy over every possible phylogenomic dataset and problem. After all, the use of 20-state amino acid recoding is usually justified on the grounds that, similarly to other recoding strategies such as Dayhoff-6, it masks substitutions (particularly at third codon positions) that tend to be saturated, compositionally heterogeneous, and phylogenetically misleading. Accordingly, if evidence exists that once translated into a set of amino acid sequences a dataset is still compositionally heterogeneous, saturated, or both, the use of a high-order recoding that more efficiently reduces saturation and compositional heterogeneity should be justified. Indeed, as suggested also by Laumer,^41^ if we accept the logic by which amino acid data are generated by recoding nucleotide data, and consider that every recoding strategy can be characterised as an approach to recode nucleotide sequences (by directly clustering codons into bins), we have to accept that standard (20-state) amino acid recoding is just one of many, related recoding strategies in which nucleotide datasets of protein coding genes can be transformed.

Dayhoff-6^33^ (Figure 1) uses a six state alphabet, and is the most widely used, high-order^41^ recoding scheme. However, promising experiments have also been completed using 9-,12-,15- and 18-state alphabets.^31^ Dayhoff-6 partitions the 20 amino acids (or their codons) into six differently sized bins: one 5-amino acid bin, two 4-amino acid bins, two 3-amino acid bins and one 1-amino acid bin, based on how frequently different amino acids substitute for each other, i.e. based on their exchangeability rates^11^ (Figure 1). Other 6-state recoding schemes have been proposed including the 6-bin recoding of Kosiol et al.^28^ – KGB6, and the 6-bin recoding scheme of Susko and Roger^29^ – SR6, see also.^11^ KGB6 and SR6 differ from Dayhoff-6 in the details of the amino acid partitioning rule they implement, but the amino acid content of their bins overlaps.^11^

When a high-order recoding (i.e. a recoding with less than 20 states) is used, substitutions among amino acids within the same bin are masked through a process of synonymization. This process is expected to reduce compositional heterogeneity and saturation because the amino acids clustered within each bin are usually identified based on the properties that they share. Amino acids within the same bin are therefore expected to interchange among each other more frequently than with amino acids from different bins. Accordingly, it is expected that within-bin substitutions should be more common than across-bin substitutions as the former are less likely to disrupt protein function^11^; Figure 1.

Our study uses simulated and empirical data (see Figure S1 for a visual summary of our experimental protocol) to investigate whether the use of 6-state recoding (hereafter only referred to as a recoding), can improve the accuracy of phylogenomic analyses. We used the Dayhoff-6 recoding scheme as this is the most common recoding in the literature, but we performed sensitivity tests using KGB6 and SR6 to evaluate whether using different 6-state recodings would have led to different conclusions.

#### Datasets

We used the dataset of Kapli and Telford^12^ which consists of 200 alignments of 30,000 sites and 97 taxa simulated under the CAT-LG + G model, assuming that either the comb jellies (Ctenophora-sister hypothesis; 100 alignments) or the sponges (Porifera-sister hypothesis; 100 alignments) are the sister of all the other animals (Figure S2). The first topology (Ctenophora-sister) represents the case when the simulation target is in the “Farris zone” (Figure S2), while the second topology (Porifera-sister) represents the case when the simulation target is in the “Felsenstein zone” (Figure S2).^12^ Having been simulated using CAT-LG + G, the alignments are across-site, rate (the Gamma component of the model^38^) and compositionally (the CAT component^23^) heterogeneous. The alignments display also heterogeneity of the amino acid replacement rate process (the LG component^56^), see^4^^,^^11^ for introductions to the heterogeneity of amino acid substitution models. However, our alignments are not across-lineage compositionally heterogeneous. This is not a problem because Dayhoff-6 reduces both across-site and across-lineage compositional heterogeneity, as well as saturation.^11^ Lack of across-lineage compositional heterogeneity in our datasets implies that our simulations only demonstrate the behavior of recodings when the datasets are across-site compositional heterogeneous. However, other studies have tested recodings on across-lineage compositionally heterogeneous datasets^29^^,^^31^^,^^32^ achieving results comparable to ours, at the least to the extent to which these studies can be compared to our own (e.g. when contrasting results from datasets of comparable size).

The alignments were subsampled to generate datasets with different numbers of characters and taxa. We generated datasets of 1,000, 5,000, 10,000, and 30,000 sites and 20 taxa; as well as datasets of 30,000 sites with 4, 10, and 20 taxa. When subsampling characters from the datasets, we made sure that each character was sampled only once in each subsampled dataset, and that all key animal lineages in the original 97-taxon trees were retained – see Figure S2 for our target topologies. Taxon subsampling was fixed so that all our 20-, 10- and 4-taxon datasets included the same terminals (Figure S2). We decided to use datasets with 20 and 4 taxa to maximise comparability with previous studies.^31^^,^^32^

#### Analyses of the simulated datasets

A total of 200 datasets of 30,000 sites and 20 taxa (100 assuming Porifera-sister to be true and 100 assuming Ctenophora-sister to be true) were analyzed using a General Time Reversible^39^ (GTR + G) model, accounting for amino acid replacement rate heterogeneity – GTR, and across-site rate heterogeneity (+G;^38^). We then used a series of nCAT models where the number of site-frequency categories was fixed *a priori* to 10, 30, 60, 120, and 240 to account for greater across-site compositional heterogeneity. These models should not be confused with the precomputed CAT models^67^ implemented in ML software such as IQtree^68^ and generally referred to as C10, C20, … Cn. This is because, despite nCAT models have a fixed number of site-frequency categories like the C10, … Cn models, nCAT models are not precomputed. The nCAT10 to nCAT240 models used a simple Poisson process (F81^37^), to model amino acid replacement rate, and a Gamma distribution (+G) to account for across-site rate heterogeneity. Accordingly, the full models used in our study were the nCAT10-F81 + G to nCAT240-F81 + G. We then used CAT-F81 + G which estimates the number of site-frequency categories during tree search, and CAT-GTR + G, which combines the properties of CAT + G and GTR + G, modeling across-site compositional and rate heterogeneity and amino acid replacement rate heterogeneity.

Datasets of 1,000 to 10,000 sites and 20 taxa, and datasets of 30,000 sites with 4 or 10 taxa were only analyzed under nCAT10 and used for tests aimed at investigating the effect of changing alignment length and taxon sampling density on our results. The nCAT10 model was selected *a posteriori* for these analyses, because it emerged from the study of the 30,000 sites and 20 taxa alignments that this model achieve the most distinguishable results when applied to our amino acid and recoded alignments.

Bayesian analyses were performed in Phylobayes.^69^ Phylobayes MPI v.18 was used to perform CAT, CAT-GTR and GTR analyses. Phylobayes-MPI-nCAT was used to perform analyses using nCAT models. Bayesian analyses of 1,000 site alignments were run for 1,000 cycles (with a burnin of 500 cycles and a subsampling frequency of 5). All other Bayesian analyses used 2,000 cycles, with a burnin of 1000 cycles and a subsampling frequency of 10. For each dataset a single run was completed, as is customary when multiple datasets are used in jackknife and similar computation intensive experiments.^18^^,^^70^ However, to make sure that 2,000 cycles were enough to reach convergence, under nCAT10, we also completed (for each dataset) two more independent runs of 10,000 cycles, testing convergence after 2,000 and 10,000 cycles (using burnins of 1,000 and 3,000 cycles respectively). In addition, to make sure that the use of Bayesian analysis did not bias our results, we performed Maximum Likelihood (ML) analyses in RAxML^71^ under GTR + G (30,000 sites and 20 taxa datasets) with 200 bootstrap replicates. For the ML analyses we used GTR + G as it is the only model, among the ones we used, with an ML implementation. All datasets were analyzed as amino acids and Dayhoff-6 recoded alignments. Analyses of the 30,000 sites and 20 taxa datasets were also performed (under nCAT10) using SR6^29^ and KGB6^28^ to test the effect of using alternative 6-state recoding schemes on our results.

#### Measuring phylogenetic accuracy

Phylogenetic accuracy could simply be defined as the inference of the target (i.e. true) tree. In our case, this would be the successful inference of trees displaying Ctenophora-sister or Porifera-sister, depending on the simulation set. However, accuracy without support is a weak and potentially misleading measure of the utility of a phylogenetic method. We thus define a tree to have been accurately resolved when the target tree is correctly inferred with support (posterior probability in Bayesian analyses and bootstrap in ML) ≥ 0.5. To make sure that using a 0.5 cutoff to define accuracy did not bias our results, under nCAT10, we also tested the effect of defining as accurate only analyses that inferred the target tree with a posterior probability ≥0.95. In addition to accurately resolved trees, we distinguished incorrect trees (where the wrong tree was inferred with support ≥0.5, or ≥0.95), and unresolved trees (where either the correct or the incorrect tree was inferred but with support<0.5, or <0.95).

#### Measuring model adequacy

Posterior Predictive Analysis (PPA) was used to evaluate whether the considered model could adequately describe the across-site compositional heterogeneity of our simulated and empirical datasets. The test statistic for these analyses is the mean diversity (PPA-Div^72^) per site (mean number of distinct states per sites), which is computed on the true data, and compared against its null (posterior predictive) distribution. In our analyses of empirical datasets, we also used PPA to test whether the considered models could adequately describe the lineage-specific compositional heterogeneity of the data. For these analyses the test statistic used was the mean deviation measured as the sum over the 20 aminoacids of the absolute differences between the taxon-specific and global empirical frequencies (PPA-Mean) – see Phylobayes manual. The observed value is computed on the true data and compared with its null (posterior predictive) distribution. PPA-Mean has not been calculated for our simulated data because these datasets, having been simulated with a model that is compositionally-homogeneous across-lineage (CAT-LG + G), are not across-lineage compositionally heterogeneous.

The ability of different models to adequately describe the compositional heterogeneity of amino acid and recoded datasets, was expressed using Z-scores, the number of standard deviations separating the heterogeneity observed for the true data from the average heterogeneity of the null (posterior predictive) distribution (Phylobayes manual and^11^). We interpret Z-scores as follows: a *Z* score value within the interval 2 to −2 indicates that the hypothesis that the model adequately describes the data cannot be rejected.^11^ However, these boundaries are set subjectively, as it is clear that the ability to describe the data of models achieving a Z-scores of 1.99 and 2.01 (for example) should not be worth distinguishing. Hence here we use PPA scores only as means to quantify the fit of the different models. As we need to discuss these models and their fit in text, we refer to models for which Z > |10| to be very poor descriptors of the heterogeneity in the data. Models for which: |5| < Z < |10| are deemed to describe the data poorly, and models for which |3| < Z < |5| are described as having a fit that varies from acceptable to fairly poor. Models with |2| < Z < |3| are deemed to be describing compositional heterogeneity in an acceptable way, and finally models for which |0| < Z < |2| adequately model the compositional heterogeneity of the data.

All PPA analyses were performed in Phylobayes MPI^69^ (2,000 cycles with a burnin of 1000 generations, subsampling every 10 generations). For CAT, GTR and CAT-GTR, we used Phylobayes MPI v.1.8. For nCAT10-nCAT20140 we used Phylobayes-nCAT.

#### Measuring saturation

To test whether recoding reduced saturation, we used PAUPv4.168^73^ to compute Neighbor Joining (NJ) trees for our amino acid and recoded 30,000 sites and 20 taxa alignments, using observed (P) distances without Gamma correction. After that, tip-to-tip distances were calculated for all the NJ trees and for the corresponding Bayesian trees (under all considered models). Saturation plots were generated, for the amino acids and recoded datasets for each considered model, by comparing corresponding tip-to-tip distances estimated from the NJ tree and the Bayesian tree. For each plot, the regression line (forced through the origin of the axes) was calculated and its gradient (M) was extracted. Saturation (Sat) was estimated (as it is customary) as Sat = 1-M.^14^^,^^74^ To test whether recoding reduced saturation, Sat scores calculated for all amino acids and recoded datasets (for all tested models) were plotted and compared.

#### Random recodings

A perl script (Recodings_generator.pl – https://bitbucket.org/bzxdp/giacomelli_et_al_2022_recodings/src/master/) was used to randomly generate recodings based on the Dayhoff-6 amino acid binning scheme: one 5-state bin, two 4-state bins, two 3-state bins and one 1-state bin. This script was used to generate 1,000 recoding schemes. The similarity of these recodings to Dayhoff-6 was then calculated using a second script (Recodings_comparator.pl – https://bitbucket.org/bzxdp/giacomelli_et_al_2022_recodings/src/master/). The latter uses intersections of sets to estimate the percent similarity between the bins of randomly generated recodings and those of a real (reference) recoding – here Dayhoff-6. This experiment found that the random recodings we generated have variable similarity to Dayhoff-6 (average 36.5%, range 0%–80%) see Table S1 - https://bitbucket.org/bzxdp/giacomelli_et_al_2022_recodings/src/master/.

We then generated 100 recodings of 0% similarity to Dayhoff-6 (0%-recodings), and 100 recodings of 90% similarity to Dayhoff-6 (90%-recodings), to evaluate whether the performance of randomly generated recodings depends on their similarity to Dayhoff-6. To generate the 0%- and 90%- recodings, we first generated 20,000,000 recodings using Recodings_generator.pl. This large number of recodings was only generated to make sure to have generated 100 recodings of 90% similarity to Dayhoff-6 as these are rarely generated (Table S1). After that, we used Recodings_comparator.pl to identify the first 100 recodings of specified (either 0% or 90%) similarity to Dayhoff-6.

Each 30,000-site and 20-taxon dataset was randomly associated with one of the randomly generated 0%- and 90%-recodings. PPA-Div scores and accuracy were calculated (under nCAT10) for the 0%-recoded and 90%-recoded datasets. These values were then compared against PPA-Div scores and accuracy values obtained using amino acids and Dayhoff-6.

#### Estimating the number of substitutions entailed by amino acid and recoded data and approximating information content

To evaluate whether there are more within Dayhoff-6 bin than across Dayhoff-6 bin substitutions, we estimated the tree length of phylogenies inferred using 0%-, 90%, and Dayhoff-6 recoded datasets. The expectation being that if there are more within-bin substitutions, as one would expect if the rationale underpinning the use of recoding is valid, Dayhoff-6 would mask more substitutions than 0%- and 90%-recodings. Accordingly, trees inferred from Dayhoff-6 recoded datasets would have a lower tree length than trees inferred using random recoding schemes. In addition, among the trees inferred using random recoding schemes, the 90%-recoding trees should be expected to have a lower tree length than the 0%-recoding trees, as the 90%-recodings are more similar to Dayhoff-6 than the 0%-recodings and should thus mask more substitutions.

The information content of an alignment can be roughly approximated by the number of substitutions it entails. To approximate the information loss incurred when recoding our simulated datasets, we calculated the tree length of our 30,000 site amino acid alignments and of their recoded version. We then expressed as a percent the difference in average tree length between the recoded and the amino acid datasets. To evaluate how the information loss was distributed across the branches of the phylogeny, we obtained branch lengths (for the simulated amino acids and recoded data) for all the branches in our target tree (this experiment was only done underPorifera-sister), and for each branch we expressed as a percent the difference in length observed when the recoded and amino acid datasets were used. These values were then mapped on the Porifera-sister tree for visual inspection. All parsimony analyses were performed using PAUPv4.168^73^ – Heuristic search with 1,000 random addition and the multree option turned off.

#### Empirical datasets

We tested three empirical datasets Whelan-D20^20^ (in text referred to as Whelan2015), Whelan-strict^19^ (in text referred to as Whelan2017), and the non-Bilateria matrix of Laumer et al.^5^ in text referred to as Laumer2019. These datasets were chosen because they addressed the phylogenetic problem represented by our simulated data. We analyzed these datasets as amino acid alignments and as 0%-recoded, 90%-recoded and Dayhoff-6 recoded datasets. All datasets were analyzed using nCAT60.^13^

The three datasets use different outgroups. To rationalise the analyses and make them more comparable, we removed all non Choanoflagellata outgroups from Whelan2015 and Laumer2019. Whelan2017 already include only choanoflagellate outgroups. To evaluate whether the information content of these datasets was comparable, we used parsimony to estimate their tree length, and divided these values by the number of sites in each dataset, approximating in this way the number of substitutions per site of each dataset. We tested whether nCAT60 adequately describes these datasets using PPA-Div and PPA-Mean (see above measuring model adequacy). As our simulated data were generated under CAT-LG + G, to make sure that results from our simulated and empirical datasets were comparable, we also tested (using PPA-Div) whether CAT-LG + G could describe the across-site compositional heterogeneity of the three empirical datasets. Support values were obtained (under nCAT60) for both Porifera-sister and Ctenophora-sister, under all recoding strategies (0%-recodings, 90%-recodings and Dayhoff-6). These values were plotted and compared against each other. We also plotted support values obtained, from our simulated datasets, when Ctenophora-sister and Porifera-sister were true and compared these values against those obtained from the empirical datasets. For the simulated datasets, we plotted the values obtained under nCAT10. This is because, with the simulated datasets, nCAT10 achieved PPA-Div scores comparable to those achieved by nCAT60 with the empirical datasets, suggesting that nCAT10 fits our simulated amino acid datasets similarly to how nCAT60 fits the empirical datasets.

#### Green phylogenomics

To estimate whether recoding can be a useful tool for green computing, we obtained the time used by Phylobayes MPI v.1.8, to complete 50 GTR, and CAT-GTR analyses for both amino acid and recoded data, using the timestamps in the trace files. All analyses were parallelised over 28 Intel Xeon Gold 6226R CPU – 2.90 GHz processors.
