# Supplementary material for: Resolving tricky nodes in the tree of life through amino acid recoding
Source: iScience. 2022 Nov 15;25(12):105594. doi: 10.1016/j.isci.2022.105594 (PMC9706708; doi:10.1016/j.isci.2022.105594)

iScience, Volume *25*

Supplemental information

Resolving tricky nodes in the tree of life through amino acid recoding

Mattia Giacomelli, Maria Eleonora Rossi, Jesus Lozano-Fernandez, Roberto Feuda, and Davide Pisani

Figure S1 – Complete experimental pipeline (see Star Methods).

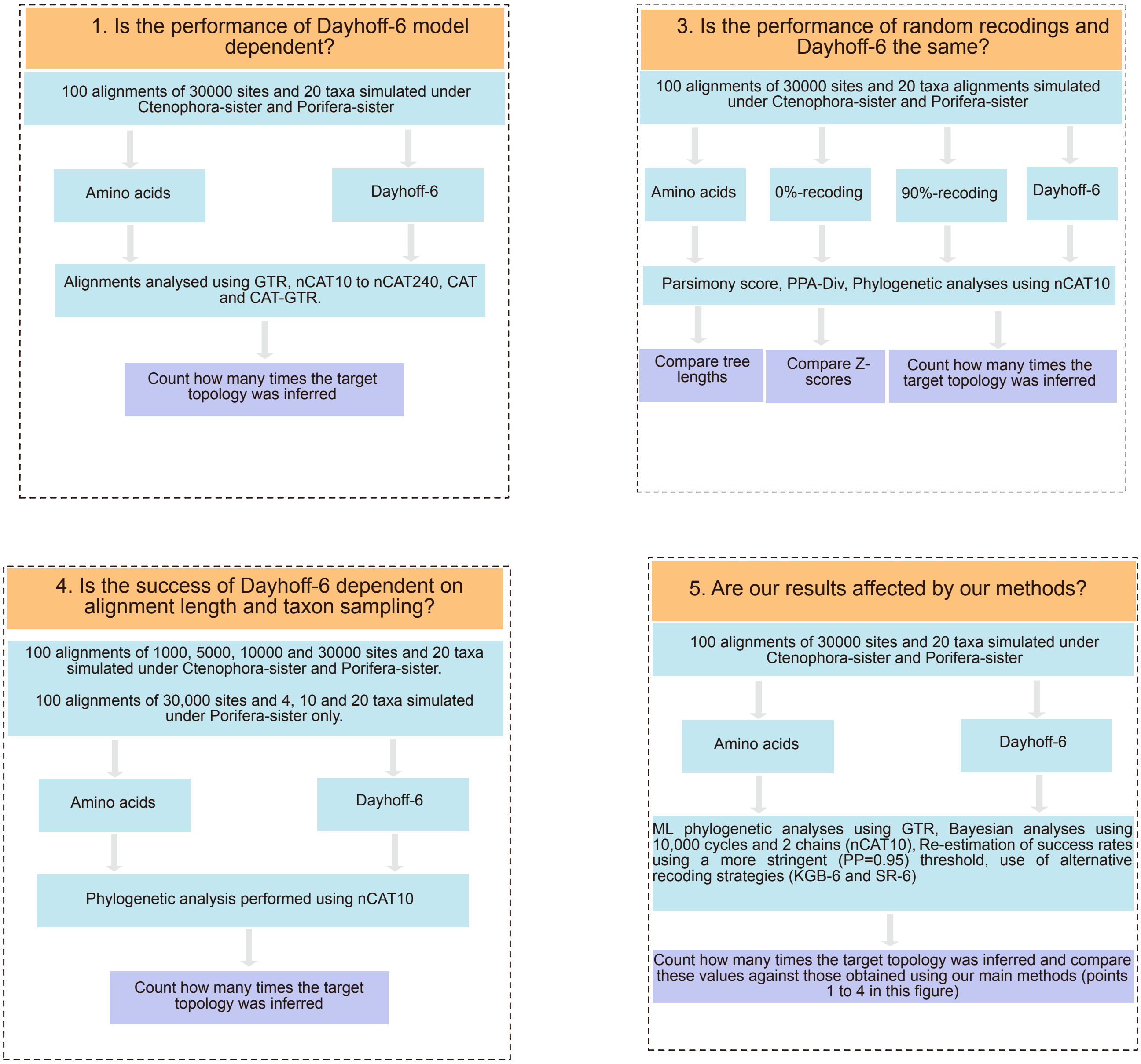

Figure S2 - The target trees of our simulations (see Star Methods)

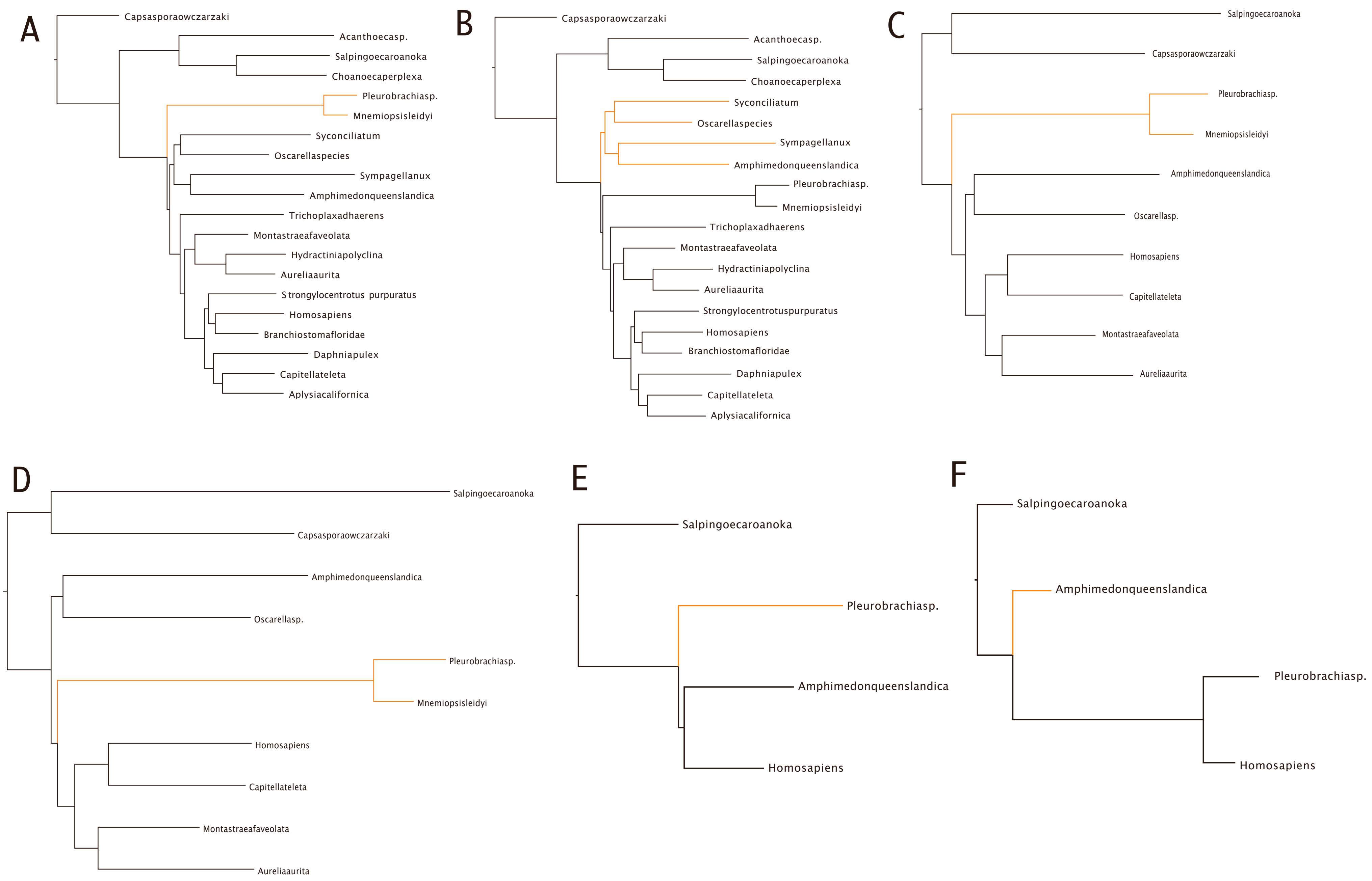

Figure S3 Recoding reduces saturation (see Fig. 2 main text).

A box plot representation of the amount of saturation identified in the amino acid (top) and recoded (bottom) data, using different substitution models. Note that more saturation is progressively identified in the amino acid alignments as more parameter rich models are used, suggesting that not even CAT-GTR can account for all the multiple substitutions that we expect to have occurred in these alignments. Differently, for the recoded data, we find that the models with 60 or more site-frequency categories identify comparable numbers of substitutions, suggesting that nCAT60 is already sufficient to account for the saturation in the recoded data. As nCAT60, nCAT120, nCAT240, CAT and CAT-GTR achieve different TA_{Rec}, we interpret this result to suggest that differences in accuracy observed (for the recoded datasets) are not primarily caused by the fact that recoding the data reduces saturation (Total number of cycles= 2,000; burnin= 1,000; subsampling frequency= 10).

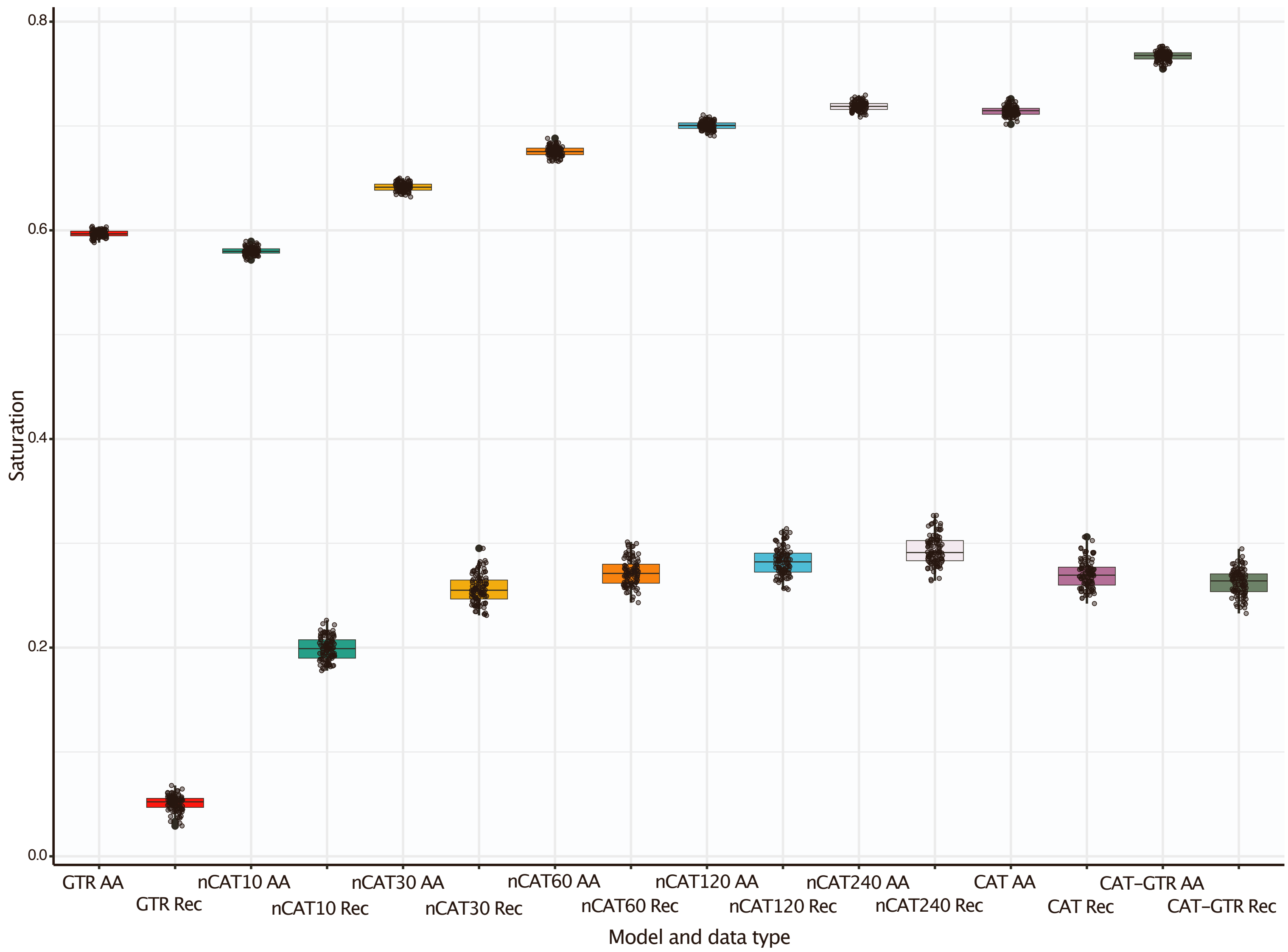

Figure S4 Results from Maximum Likelihood and Bayesian analyses are indistinguishable (see Fig. 2 main text).

Comparison of results obtained under GTR, when amino acid and Dayhoff-6 datasets are analysed using Bayesian analysis or Maximum Likelihood. For the ML analyses we defined a tree to be accurate if the target tree was inferred with a minimal bootstrap support of 50% (Bootstrap calculated using 200 replicates – see Star Methods). Note that the comparison of ML and Bayesian analysis could only be done under GTR as the nCAT models, CAT and CAT-GTR are not implemented in ML software. Results show that the exact same result is obtained for GTR under both ML and Bayesian analysis. In Green: Correctly inferred trees. Dark Orange: Incorrect trees.

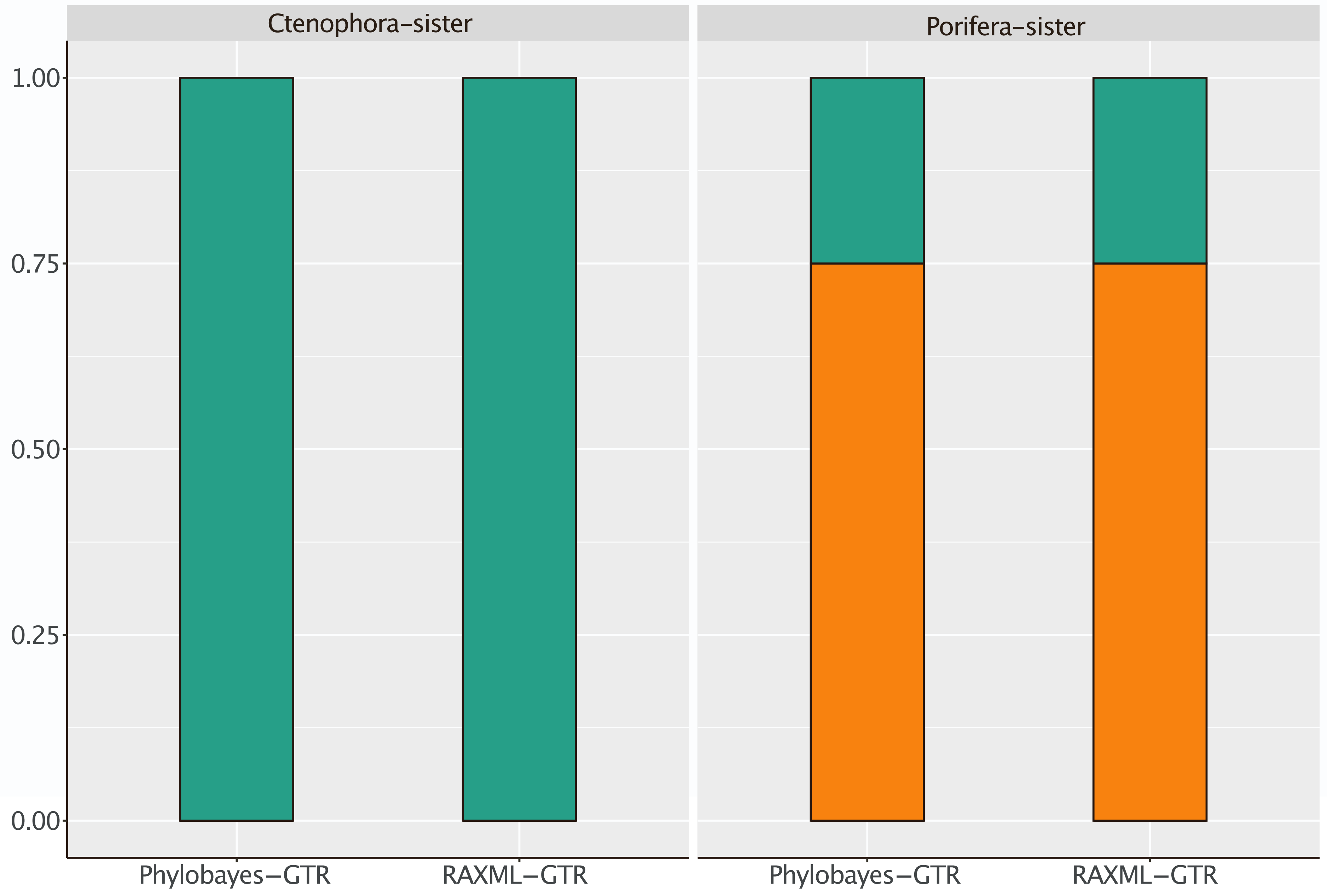

Figure S5 Increasing the number of generations and burnin in our Bayesian analyses does not impact our results (see Fig. 2 main text).

Comparison of the accuracy of amino acids and recoded datasets (under nCAT10), as the total number of cycles is incremented from 2,000 to 10,000, and one or two runs are used. Convergence was already achieved after 2,000 cycles using a burn in of 1,000 generations, accordingly, results did not change as we increased the number of cycles to 10,000 and the number of runs to two. In Green: Correct trees; Dark Orange: Incorrect Trees; Light Orange: Uncertain trees.

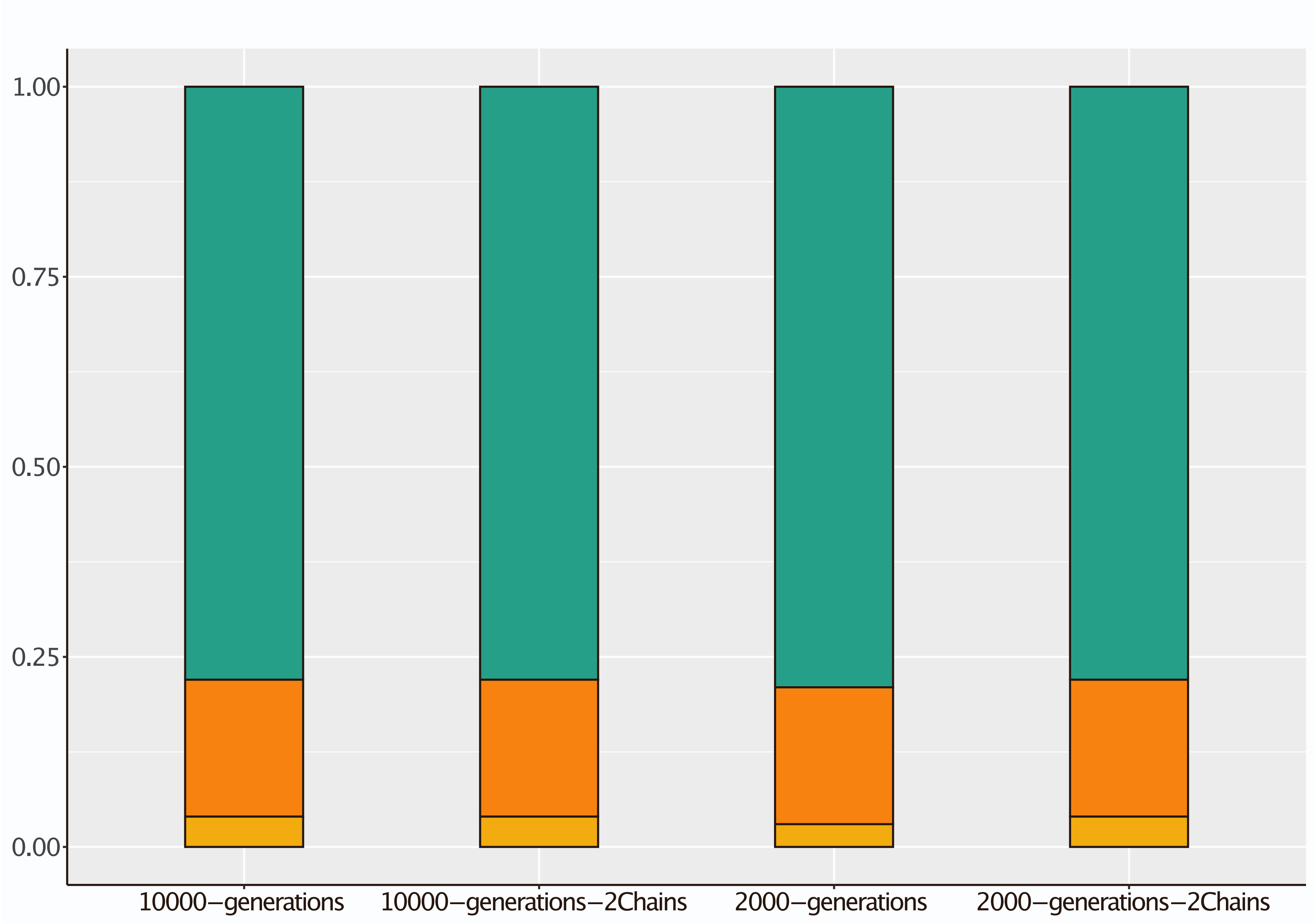

Figure S6 Using a more stringent threshold to define accuracy does not impact our conclusions (see Fig. 2 main text)

A comparison of the accuracy of amino acids and recoded datasets when the support threshold used to define an accurate result is increased to PP= 0.95. Analyses performed under nCAT10 in Phylobayes (Total number of cycles= 2,000; burnin= 1,000; subsampling frequency= 10). In Green: Correct trees; Dark Orange: Incorrect Trees; Light Orange: Uncertain trees.

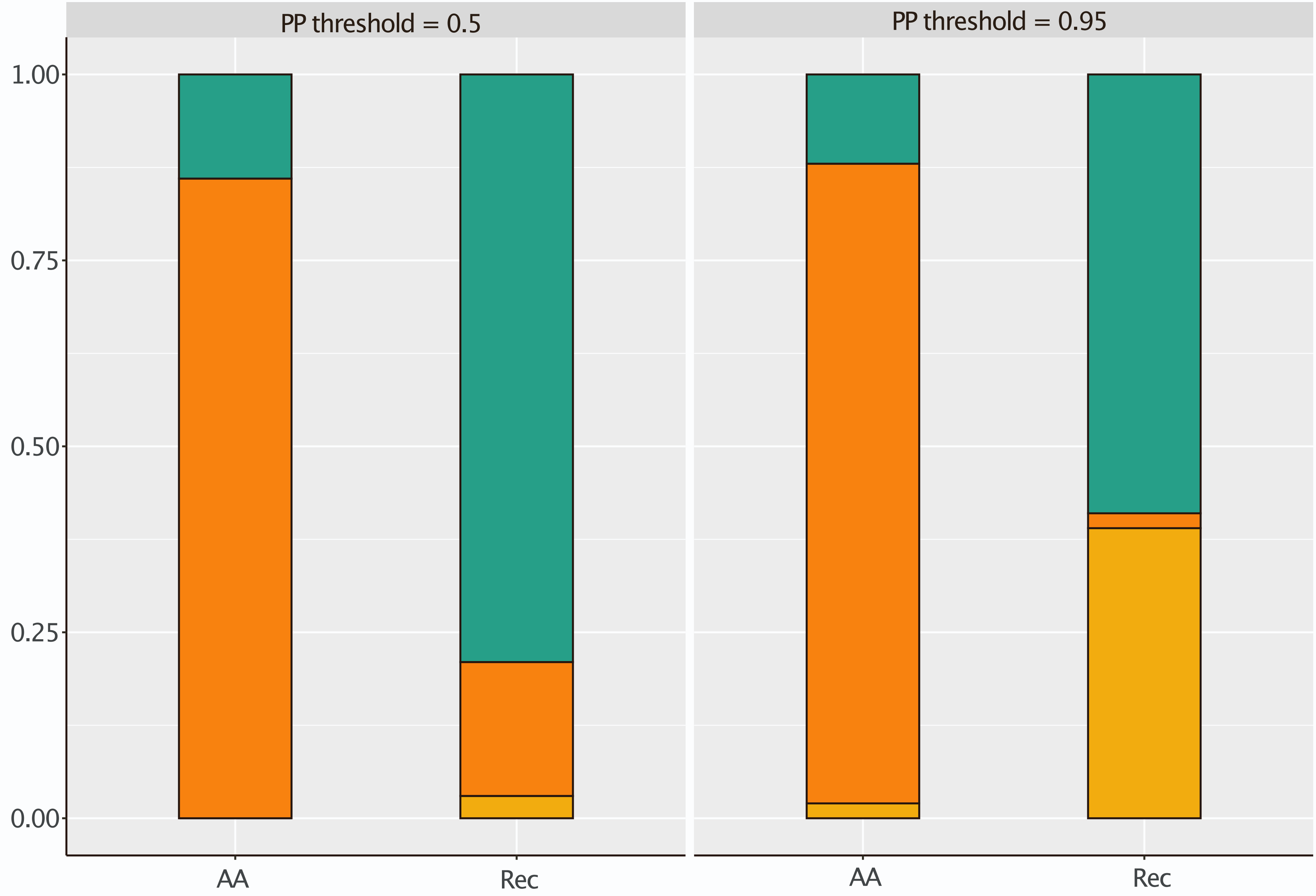

Figure S7 Using alternative recoding strategies does not impact our conclusions (see Fig. 2 main text).
A comparison of the accuracy of amino acids and recoded data sets when SR6 or KGB6 are used instead of Dayhoff-6. Analyses performed under nCAT10 in Phylobayes (Total number of cycles= 2,000; burnin= 1,000; subsampling frequency= 10). This analysis shows that SR6 performs slightly better than Dayhoff-6 and KGB6 slightly worse. However, the results are fully comparable. In Green: Correct trees; Dark Orange: Incorrect Trees; Light Orange: Uncertain trees.

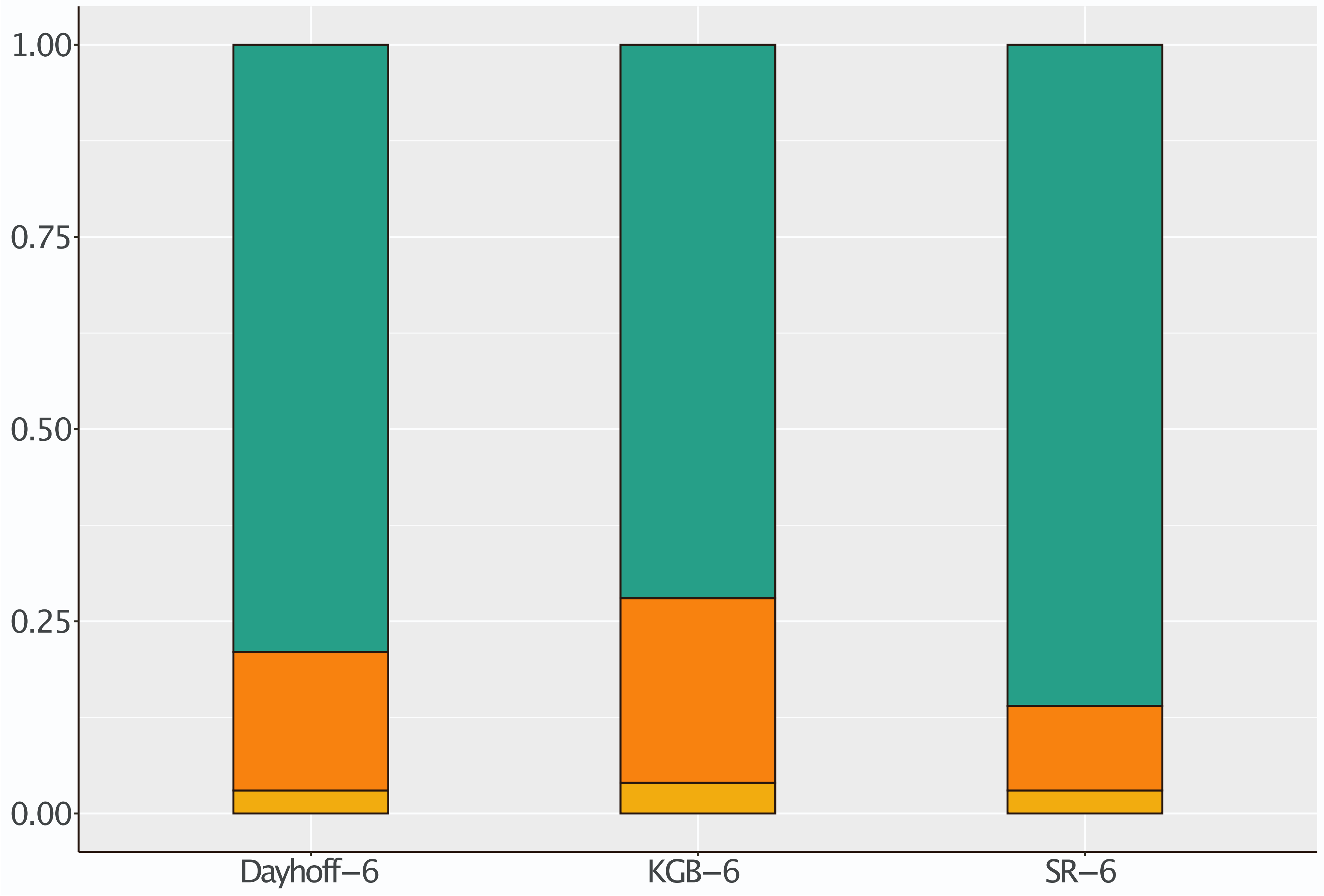

Figure S8 A graphical representation of the similarity between the 0%-, 90%- and Dayhoff-6 recoded versions of the same amino acid alignment (see Fig. 1 and Star Methods)

The alignment represents the first 26 sites of a randomly chosen simulated dataset. The figure shows that there is a clear similarity between the various coding strategies. Random recodings do not scramble the signal in the data, they randomly select which substitutions to mask. The figure shows that Dayhoff-6 masks more substitutions than random recodings. Furthermore, the figure shows that as the recoding become more similar to Dayhoff-6, more substitutions are masked (see also main text). Note that, in random recodings, amino acids are randomly reassigned to bins, so a common naming system for the bins of different random recodings cannot be achieved in this figure.Hence in the figure we only use colours. If at a site, all species have the same state (either all species have the same amino acid, or all amino acids observed at a site belong to the same bin), we use a single colour to represent the site. If two states are present (either two amino acids or two bins) we use two colours and so on. Across sites, the same colour was used for sites having the same number of states.

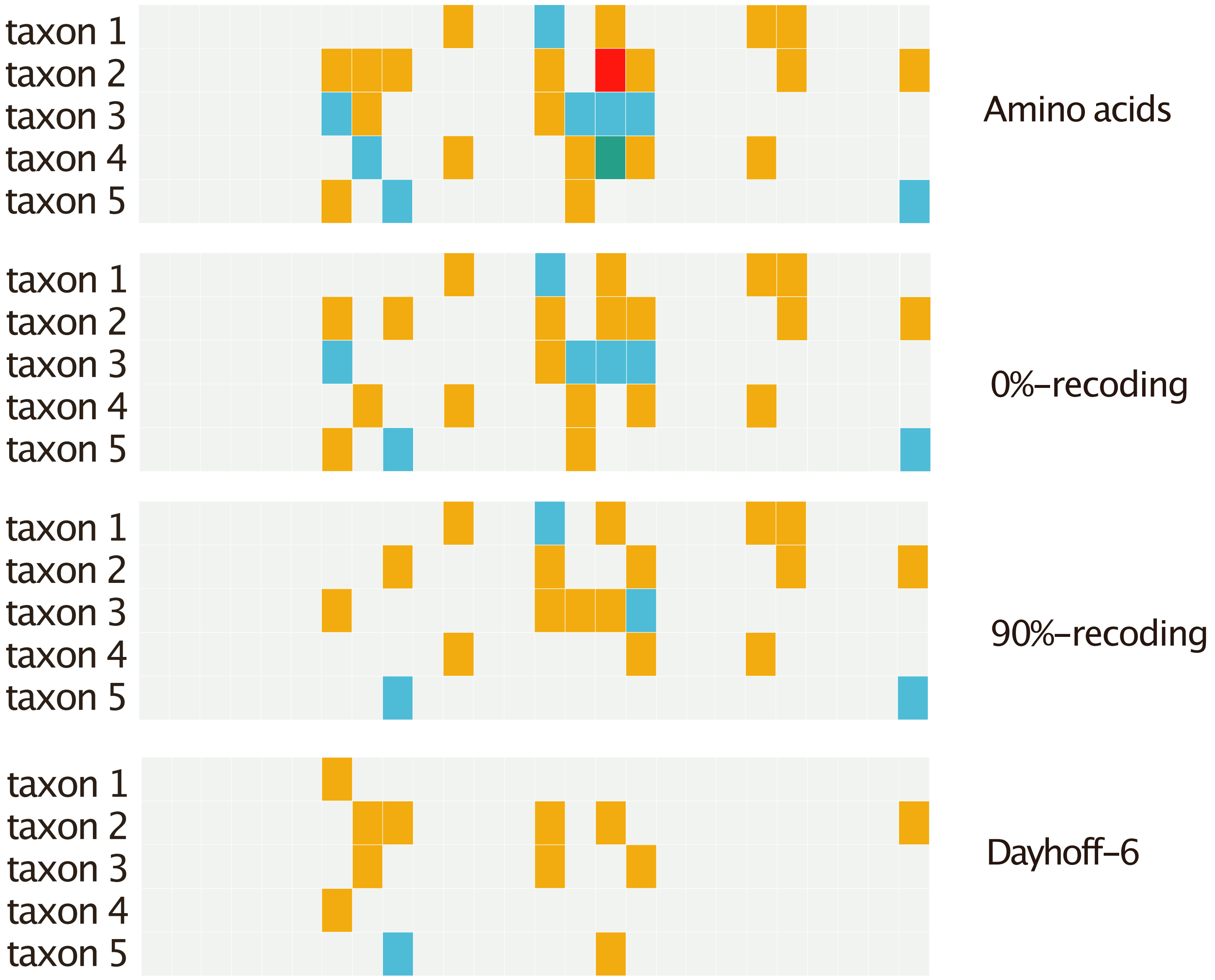

Figure S9 Proportion of substitutions masked by Dayhoff-6 across the branches of the target topology (see Fig. 2 and Fig 4).

This analysis has been performed using datasets simulated under Porifera-sister only. The tree shows that the proportion of substitutions masked is fairly homogeneous across the tree, even if there seem to be a little more masked substitutions towards the tip of the tree. Note: the tree is unrooted.

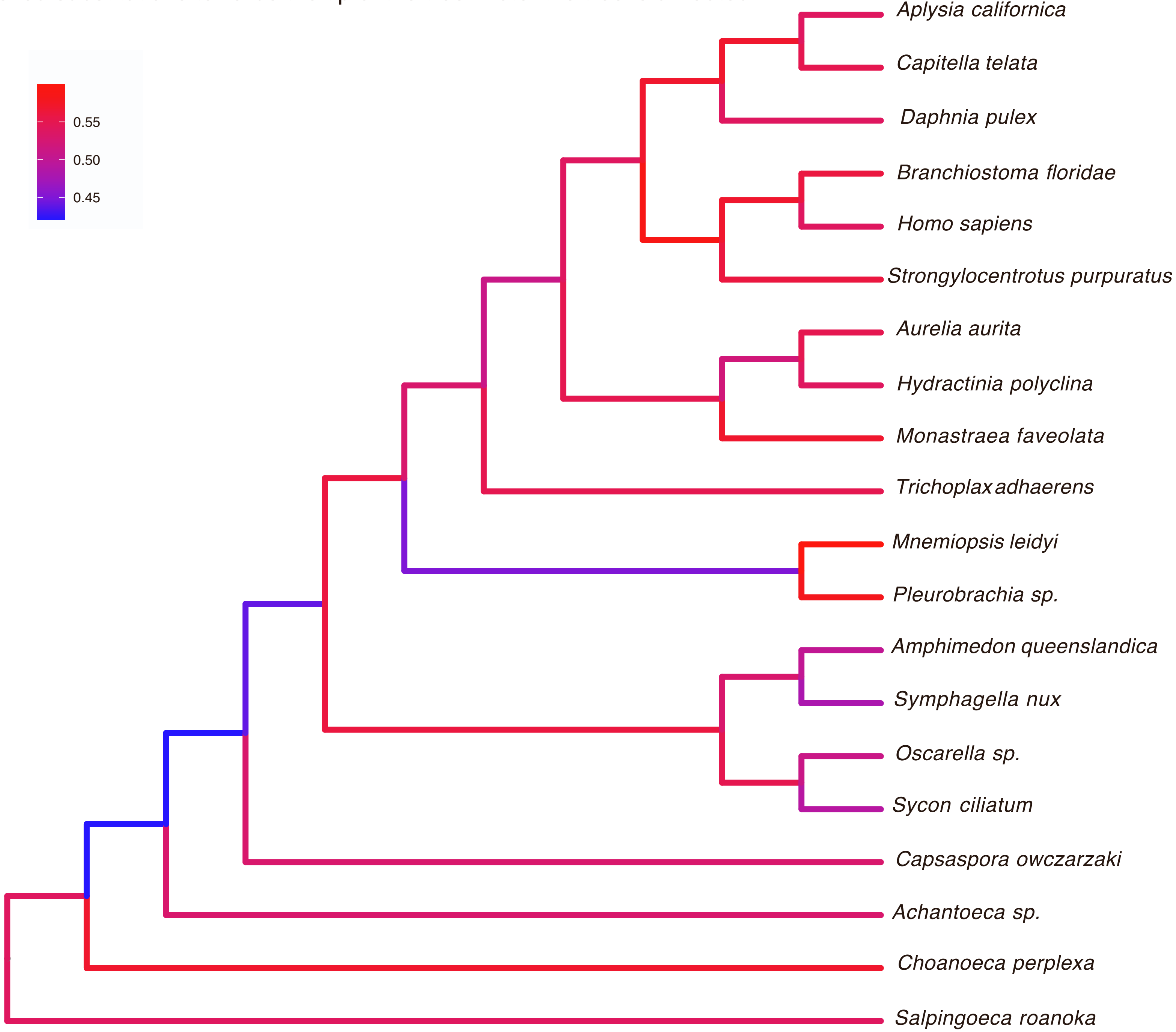

Figure S10 Accuracy of amino acid and recoded datasets as taxon sampling increases from 4 to 10 and 20 species (see Fig. 2 and Fig. 5).

Our results suggest that the accuracy of analyses of recoded data increases with the number of taxa. This result follows expectations based on previous knowledge from theoretical phylogenetics [43]. Analyses performed under nCAT10 in Phylobayes (Total number of cycles= 2,000; burnin= 1,000; subsampling frequency= 10). In Green: Correct trees; Dark Orange: Incorrect Trees; Light Orange: Uncertain trees.

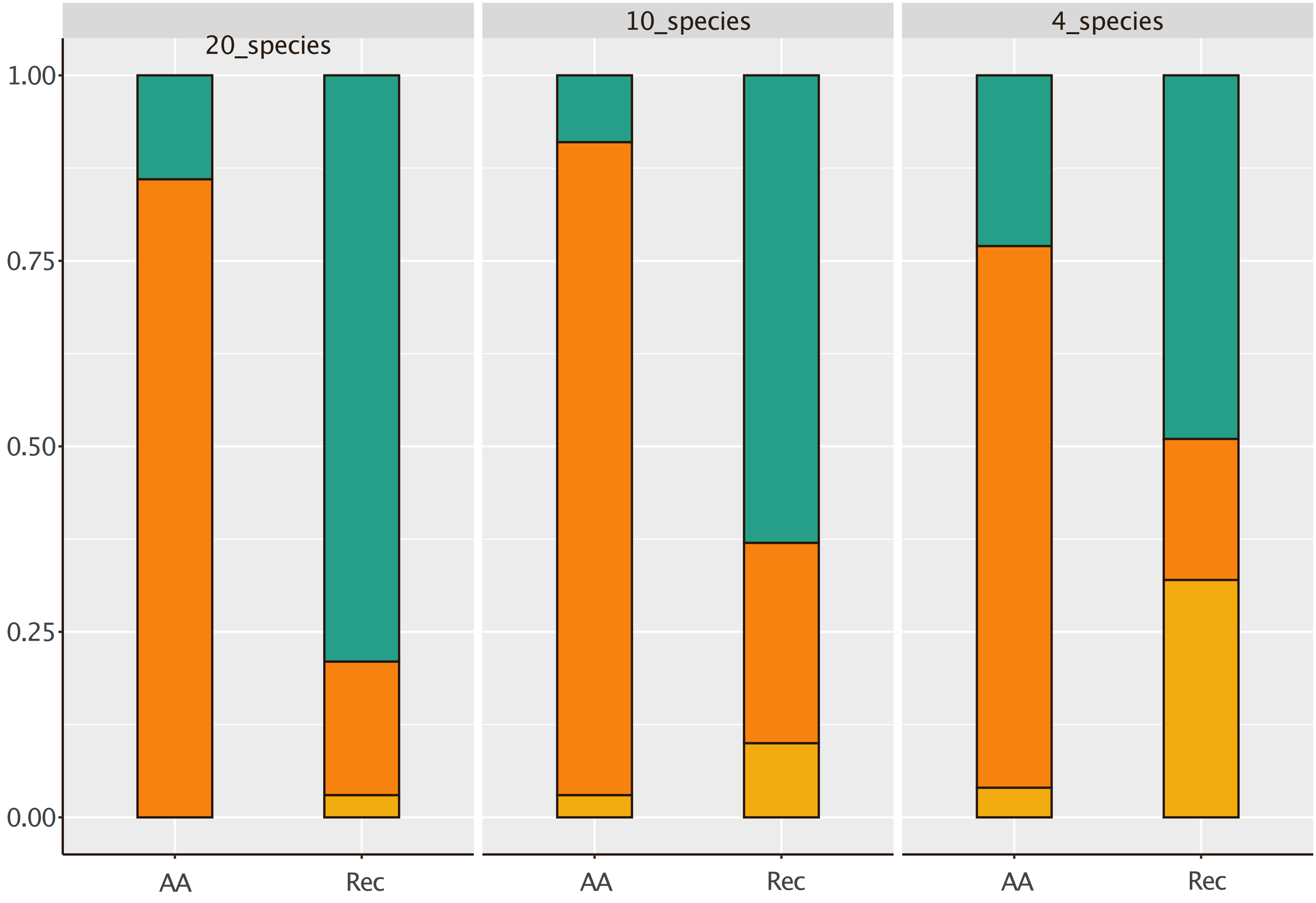

Supplement: Document S1. Figures S1–S10 [file mmc1.pdf]
